# Gut microbiota in dementia with Lewy bodies

**DOI:** 10.1038/s41531-022-00428-2

**Published:** 2022-12-09

**Authors:** Hiroshi Nishiwaki, Jun Ueyama, Kenichi Kashihara, Mikako Ito, Tomonari Hamaguchi, Tetsuya Maeda, Yoshio Tsuboi, Masahisa Katsuno, Masaaki Hirayama, Kinji Ohno

**Affiliations:** 1 Division of Neurogenetics, Center for Neurological Diseases and Cancer, Nagoya University Graduate School of Medicine, Nagoya, Japan; 2 Department of Pathophysiological Laboratory Sciences, Nagoya University Graduate School of Medicine, Nagoya, Japan; 3 Department of Neurology, Okayama Kyokuto Hospital, Okayama, Japan; 4 Okayama Neurology Clinic, Okayama, Japan; 5 Division of Neurology and Gerontology, Department of Internal Medicine, School of Medicine, Iwate Medical University, Iwate, Japan; 6 Department of Neurology, Fukuoka University, Fukuoka, Japan; 7 Department of Neurology, Nagoya University Graduate School of Medicine, Nagoya, Japan

**Keywords:** Parkinson's disease, Epidemiology, Dementia

## Abstract

Gut microbiota and fecal bile acids were analyzed in 278 patients with α-synucleinopathies, which were comprised of 28 patients with dementia with Lewy bodies (DLB), 224 patients with Parkinson’s disease (PD), and 26 patients with idiopathic rapid eye movement sleep behavior disorder (iRBD). Similarly to PD, short-chain fatty acids-producing genera were decreased in DLB. Additionally, *Ruminococcus torques* and *Collinsella* were increased in DLB, which were not changed in PD. Random forest models to differentiate DLB and PD showed that high *Ruminococcus torques* and high *Collinsella*, which presumably increase intestinal permeability, as well as low *Bifidobacterium*, which are also observed in Alzheimer’s disease, were predictive of DLB. As *Ruminococcus torques* and *Collinsella* are also major secondary bile acids-producing bacteria, we quantified fecal bile acids and found that the production of ursodeoxycholic acid (UDCA) was high in DLB. Increased UDCA in DLB may mitigate neuroinflammation at the substantia nigra, whereas neuroinflammation may not be critical at the neocortex. Theraeutic intervention to increase *Bifidobacteirum* and its metabolites may retard the development and progression of DLB.

## Introduction

α-Synucleinopathies are a group of neurodegenerative disorders characterized by abnormal aggregation of α-synuclein fibrils (Lewy bodies) in the brain, and is comprised of iRBD, PD, and DLB^1^. Multiple system atrophy (MSA) is attributed to another species of abnormal aggregation of α-synuclein fibrils^2^, and will not be addressed in this communication. More than 90% of iRBD patients develop other forms of α-synucleinopathies in ten or more years^3^. PD patients develop motor symptoms without dementia at first. Some PD patients later develop dementia, which is called PD dementia (PDD)^4^. In contrast, DLB patients develop dementia before or less than one year after the onset of motor symptoms^5^. DLB is a type of dementia characterized by visual hallucinations, fluctuating cognitive impairment, sleep disturbance, movement disorders (parkinsonism), and autonomic dysfunctions^5,6^. DLB accounts for about twenty percent of dementia and is the second most common dementia after Alzheimer’s disease^5,6^. The signs, symptoms, and cognitive profiles of PDD are similar to those of DLB^7^, and there is no essential difference in the pathology of autopsied cases, but unidentified factor(s) should differentiate DLB and PDD. Gut microbiota could be one of the differentiating factors. In α-synucleinopathies, Lewy bodies are observed in the lower brainstem, the cerebral cortex^8^, the olfactory bulb^9^, the salivary glands^10^, the skin^11^, the autonomic nervous system^12^, and the intestine^10,13,14^. In 2003, Braak proposed a hypothesis that abnormal α-synuclein fibrils start from the nucleus tractus solitarius of the vagal nerve and gradually ascend to the substantia nigra^9,15–17^. PD patients sometimes develop constipation, iRBD, and depression about 20, 10, and 5 years before the onset of motor symptoms^18^, which is in accordance with Braak’s hypothesis.

There are more than 20 studies on gut microbiota in patients with PD and iRBD reported by us^19–22^ and others^23–40^, but gut microbiota in DLB has not been reported to the best of our knowledge. We previously showed by meta-analysis of gut microbiota in different countries that mucin-degrading genus *Akkermansia* was increased in PD and iRBD, while short chain fatty acids (SCFA)-producing genera *Faecalibacterium* and *Roseburia* were decreased in PD but not in iRBD^20,21^. In this study, we analyzed gut microbiota in DLB, which was compared with controls, iRBD, and PD with or without cognitive decline.

## Results

### Analysis of each taxon between controls and DLB, and controls and PD

**Fig. 1:**
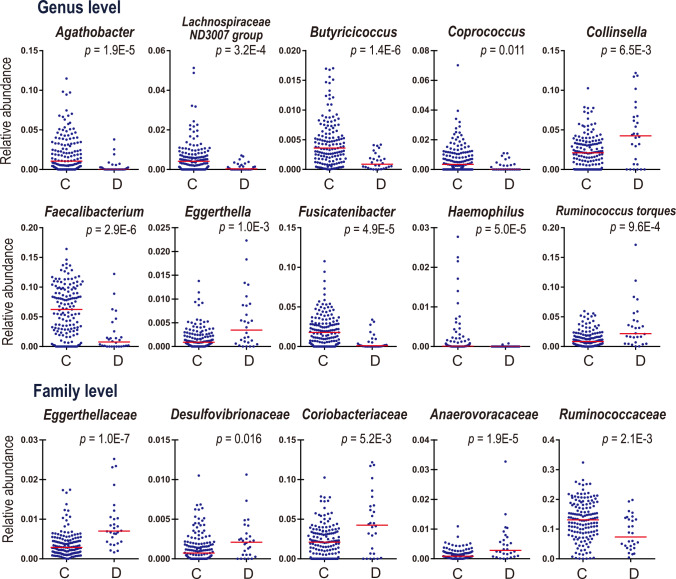
Plots of ten genera and five families that were significantly changed between controls (C) and DLB (D). Medians are indicted by red bars. *P*-values are calculated by Wilcoxon rank sum test. *Q*-values by the Benjamini-Hochberg method are indicated in Supplementary Table 2a at the genus level and 2b at the family level.

We obtained fecal samples in 224 PD patients, 26 iRBD patients, 28 DLB patients, and 147 controls. The numbers of PD patients at Hoehn & Yahr stages 1 to 5 with or without dementia are indicated in Supplementary Table 1. The collation of the demographic and clinical features between (i) controls and DLB, (ii) controls and PD, and (iii) controls and iRBD is indicated in Table 1. Five to six features out of the seven collated features were statistically different in either DLB, PD, or iRBD compared to controls. Next, we examined taxonomic differences between controls and DLB using Analysis of Compositions of Microbiomes with Bias Correction (ANCOM-BC), which examines taxonomic differences in two groups^41^, and Wilcoxon rank sum test (Supplementary Table 2a at the genus level and 2b at the family level). In ANCOM-BC, five confounding factors (age, sex, BMI, constipation, and PPI) were included in the analysis. In DLB, at the genus level, three genera were increased (*Collinsella*, *Eggerthella*, and *Ruminococcus torques*) and seven genera were decreased (*Agathobacter*, *Lachnospiraceae ND3007 group*, *Butyricicoccus*, *Coprococcus*, *Faecalibacterium*, *Fusicatenibacter*, and *Haemophilus*) after adjusting for the confounding factors (Fig. 1 and Supplementary Table 2a). In DLB, at the family level, four families were increased (*Eggerthellaceae*, *Desulfovibrionaceae*, *Coriobacteriaceae*, and *Anaerovoracaceae*) and one family was decreased (*Ruminococcaceae*) after adjusting for the confounding factors (Fig. 1 and Supplementary Table 2b). Nested cross-validation of random forest models to differentiate controls and DLB gave rise to the area under the receiver operating characteristic curve (AUROC) of 0.816 (95% confidence interval: 0.714–0.917) (Supplementary Fig. 1), indicating that gut bacteria were able to differentiate controls and DLB efficiently. Fifteen genera made the maximum AUROC by leave-one-out cross-validation in recursive feature elimination (Supplementary Fig. 1 and Supplementary Table 4). The three genera (*Collinsella*, *Eggerthella*, and *Ruminococcus torques*) that were significantly increased in ANCOM-BC and Wilcoxon rank sum test were also essential determinants in random forest models.

**Table 1:** Clinical and demographic features of controls, DLB, PD, and iRBD patients.

	Control (*n* = 147)^a^	DLB (*n* = 28)^a^		PD (*n* = 224)^a^		iRBD (*n* = 26)^a^	
			*P*-value^b^		*P*-value^b^		*P*-value^b^
Age (years)	68.3 ± 9.9	77.5 ± 5.9	5.0E-6^*^	68.2 ± 8.6	0.93	74.5 ± 6.4	2.3E-3^*^
# Females	69	14	0.84	130	0.043^*^	6	0.031^*^
Body mass index (BMI)	22.9 ± 3.0	20.9 ± 3.5	2.9E-3^*^	21.7 ± 3.0	1.9E-4^*^	24.4 ± 2.4	0.016^*^
# Constipation (≤ twice a week)	6	12	3.6E-7^*^	80	3.3E-14^*^	9	2.8E-5^*^
Stool frequency/week	7.9 ± 4.3	4.8 ± 4.6	7.7E-4^*^	4.7 ± 4.1	6.8E-12^*^	5.8 ± 5.7	0.034^*^
Disease duration (years)	–	2.1 ± 2.2	–	7.5 ± 6.1	–	6.4 ± 4.8	–
# Proton pump inhibitor	12	6	0.045^*^	35	0.038^*^	7	0.011^*^
# H_2_ blocker	6	3	0.16	8	0.79	1	1.00
MDS-UPDRS	–	59.2 ± 36.2	–	50.1 ± 23.1	–	7.6 ± 5.5	–
MDS-UPDRS III	–	31.0 ± 21.0	–	26.4 ± 13.5	–	2.0 ± 2.6	–

^a^Mean and SD are indicated when applicable. ^b^Either Student’s *t*-test or Fisher’s exact test is applied to be compared to controls. *P < 0.05.

We previously analyzed almost identical fecal samples in controls and PD using ANCOM^42^ and Wilcoxon rank sum test^20^. We then analyzed confounding factors in 18 genera and 5 families with generalized linear modeling (GLM)^20^. Here, we compared controls and PD using ANCOM-BC by simultaneously adjusting for the five confounding factors. We previously showed that eight genera (*Christensenellaceae R-7 group, Ruminococcaceae_anonymous, UBA1819, Oscillibacter, Family XIII_anonymous, Alistipes, Akkermansia*, and *Family XIII AD3011 group*) were increased in PD (see Supporting Information Fig. S2 in our previous report^20^), whereas only two genera, *Akkermansia* and *Oscillibacter*, which were a subset of the eight previous genera, were increased in our current analysis (Supplementary Table 3a). Similarly, we previously showed that seven genera (*Fusicatenibacter, Butyricicoccus, Lachnospiraceae ND3007 group, Faecalibacteriumb, Roseburia, Blautia*, and *Ruminococcaceae UCG-013*) were decreased in PD (see Supporting Information Fig. S2 in our previous report^20^), while four previous genera (*Butyricicoccus, Blautia, Fusicatenibacter*, and *Lachnospiraceae ND3007 group*) and three new genera (*Coprococcus, Monoglobus*, and *Agathobacter*) were decreased in our current analysis (Supplementary Table 3a). We previously concluded by additionally performing meta-analysis of gut microbiota in PD in five countries that PD patients had increased *Akkermansia* and decreased SCFA-producing genera^20^. The changes in these genera were indeed shared between our previous and current analyses. Nested cross-validation of random forest models to differentiate controls and PD, which were not generated in our previous report^20^, yielded the AUROC of 0.762 (0.714–0.810) (Supplementary Fig. 2). Twenty-five genera made the maximum AUROC by leave-one-out cross validation in recursive feature elimination (Supplementary Fig. 2 and Supplementary Table 5).

When DLB and PD were compared, five out of the seven decreased genera in DLB (*Agathobacter*, *Lachnospiraceae ND3007 group*, *Butyricicoccus*, *Coprococcus*, and *Fusicatenibacter*) were also decreased in PD, whereas none of the three increased genera in DLB were increased in PD. Random forest modeling showed that gut bacteria differentiated controls and PD less efficiently than controls and DLB, which was likely due to a broad spectrum of disease severities in PD compared to those in DLB.

### Analysis of the overall composition of gut microbiota

We performed PERMANOVA to examine the overall composition of gut microbiota in controls and DLB (Table 2). The overall composition of gut microbiota between controls and DLB was statistically different by all three distance metrics (Table 2a). We also found that age, sex, and PPI affected the overall composition of gut microbiota (Table 2b). Donepezil and memantine, both of which were used to treat dementia, did not affect the overall composition of gut microbiota in DLB patients (Table 2c). PERMANOVA analyses between controls and PD^20^ and between controls and iRBD^21^ were performed previously using almost the same samples, and were not repeated in this communication.

**Table 2:** PERMANOVA to examine the effect of each factor on the overall bacterial composition.

	# DLB patients	# Controls	*P*-value		
			Chao	Weighted UniFrac	Unweighted UniFrac
**(a)**	28	147			
DLB			1.0E-7^*^	1.0E-7^*^	1.20E-04^*^
**(b)**	28	142^a^			
DLB			1.0E-6^*^	1.0E-6^*^	1.3E-4^*^
Age			4.8E-3^*^	1.3E-3^*^	2.8E-3^*^
Sex			0.045^*^	0.015^*^	0.15
BMI			0.54	0.43	0.47
Constipation			0.13	0.43	0.52
PPI			1.2E-3^*^	^*^2.6E-4^*^	0.12
**(c)**	28	–			
Donepezil			0.88	0.92	0.13
Memantine			0.11	0.20	0.10

*P*-values of three distance metrics (Chao, weighted-UniFrac, and unweighted-UniFrac) are indicated. **(a)** Analysis of the effect of DLB on the overall microbial composition without considering other covariates in DLB and controls. **(b)** Analysis of the effects of DLB, age, sex, BMI, constipation, and PPI on the overall microbial composition in DLB and controls. **(c)** Analysis of the effects of donepezil and memantine, drugs for dementia, on the overall microbial composition in DLB.

### PCoA analysis, as well as integrated topological analysis with *tmap* for simultaneous mapping of the overall gut microbiota, disease states, and clinical features

**Fig. 2:**
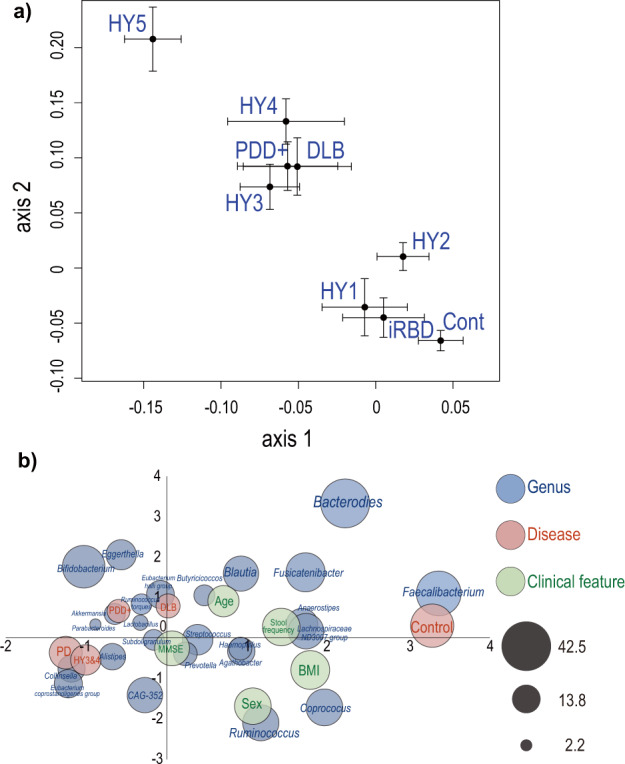
PCoA and *tmap* plots. **a** PCoA plot showing the centers of gravity and the standard errors of the overall compositions of gut microbiota in nine disease states. Bray-Curtis distance was used as a distance metric. **b** An integrated topological map, *tmap*, showing how close genera, disease states, and clinical features are to each other. Blue, red, and green circles indicate genera, disease states, and clinical features, respectively. The size of circles indicates the SAFE score, which represents the network-level association of a target feature and is used as an effect size.

PCoA to examine the difference in the overall composition of gut microbiota revealed that the centers of gravity were shifted from the lower right to the upper left with the disease progression in PD, and that the center of gravity in DLB was close to those in Hoehn & Yahr stages 3 and 4 in PD (HY3&4) and PD with Mini-Mental State Examination (MMSE) < 26 (PD with cognitive decline, PDD+) (Fig. 2a). Next, we performed *tmap*^43^ to examine the relationship between taxonomic abundances, disease states, and clinical features in the same dimensions. The *tmap* analysis revealed that controls were closely located to SCFA-producing genera (*Faecalibacterium*, *Coprococcus*, *Anaerostipes*, *Lachnospiraceae ND 3007 group*, and *Fusicatenibacter*), indicating that controls were rich in SCFA-producing genera (Fig. 2b). In addition, DLB was closely located to PDD+ and HY3&4 (Fig. 2b), which was in accordance with the PCoA analysis (Fig. 2a).

### Random forest models to differentiate DLB and HY3&4, as well as DLB and PDD+

**Fig. 3:**
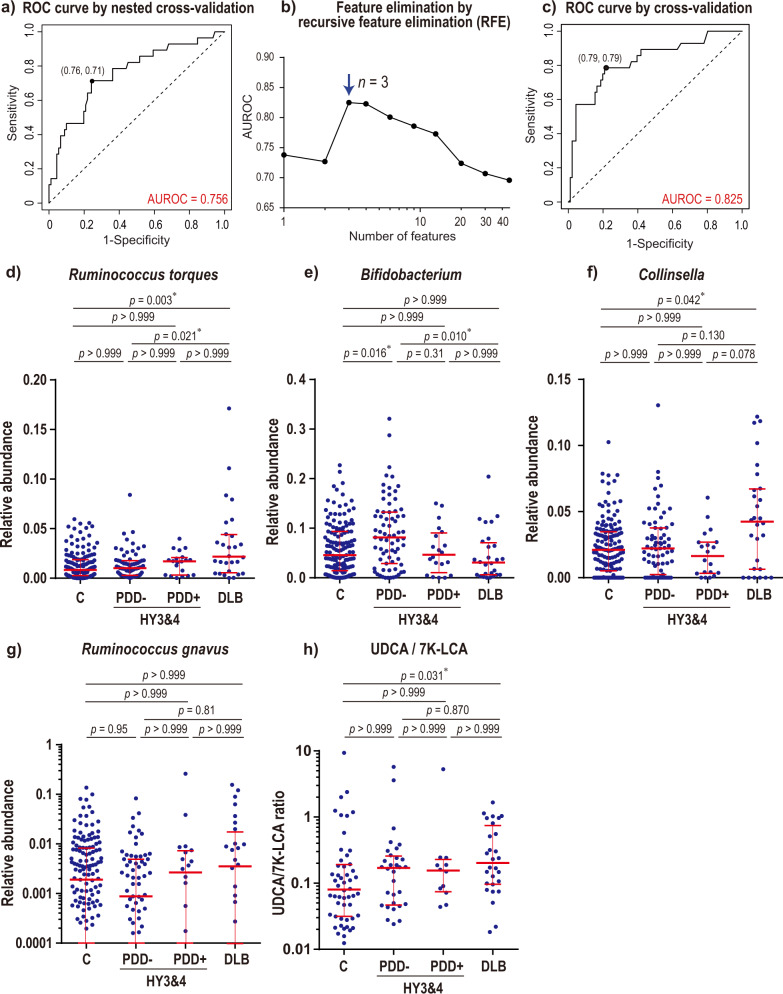
Random forest models and essential intestinal genera to differentiate DLB and Hoehn & Yahr stages 3 and 4 (HY3&4) including both PDD− and PDD+ , and plots of fecal bile acids. **a** ROC curves of nested cross-validation of random forest models to differentiate DLB and HY3&4 (both PDD− and PDD+). The optimal point by Youden index is indicated by a dot with the specificity and sensitivity in parentheses. **b** AUROCs by leave-one-out cross-validation of random forest models while genera were recursively eliminated. An arrow points to the maximum AUROC with the number of genera. The top ten genera that differentiated DLB and HY3&4 (both PDD− and PDD+), as well as the exact AUROC values, are indicated in Supplementary Table 6. **c** ROC curves of leave-one-out cross-validation of random forest models generated with three genera indicated by an arrow in **b**. The optimal point by Youden index is indicated by a dot with the specificity and sensitivity in parentheses. **d**, **e**, **f** Relative abundances of three genera indicated by an arrow in **b** in controls (*n* = 147), HY3&4 with MMSE ≥ 26^116^ (PDD−; *n* = 71), HY3&4 with MMSE < 26^116^ (PDD+ ; *n* = 20), a*n*d DLB (*n* = 28). *P*-values by Kruskal-Wallis test were all less than 0.05. *P*-values by Dunn’s post hoc test are indicated with an asterisk for *p* < 0.05. **g** Relative abundance of *Ruminococcus gnavus*, which also produces ursodeoxycholic acid (UDCA) from 7-ketolithocholic acid (7K-LCA), in the four categories. Although *p*-value by Kruskal-Wallis test was 0.40, *p*-values by Dunn’s post hoc test are indicated. Note that relative abundance is plotted on a logarithmic scale to clearly indicate medians and interquartile range. **h** Fecal UDCA/7K-LCA ratios in the four categories. *P*-value by Kruskal-Wallis test was 0.044. *P*-values by Dunn’s post hoc test are indicated with an asterisk for *p* < 0.05. UDCA and 7K-LCA were randomly measured in available fecal samples. (**d**, **e**, **f**, **g**, **h**) Median and interquartile range are indicated in red.

According to PCoA and *tmap*, the overall composition of gut microbiota in DLB was similar to those of HY3&4 and PDD+ . In order to identify bacteria that were uniquely changed in DLB, we made random forest models to differentiate DLB (*n* = 28) and HY3&4 (*n* = 91) (including both PDD− and PDD+), as well as DLB (*n* = 28) and PDD+ (*n* = 31) (including all HY stages). The AUROC to differentiate DLB and HY3&4 was 0.756 (95% confidence interval: 0.649–0.864) (Fig. 3a) by nested cross-validation. Three genera (*Ruminococcus torques*, *Bifidobacterium*, and *Collinsella*) made the maximum AUROC by leave-one-out cross-validation in recursive feature elimination (Fig. 3b). The top ten genera remained in recursive feature elimination are indicated in Supplementary Table 6. We analyzed taxonomic differences between DLB and HY3&4 by ANCOM-BC and Wilcoxon rank sum test (Supplementary Table 7a). Wilcoxon rank sum test showed that *Ruminococcus torques*, *Bifidobacterium*, and *Collinsella* were ranked first, third, and seventh, respectively. None of the 94 analyzed genera, however, were significantly changed after being corrected for multiple comparisons.

In contrast to a model to differentiate DLB and HY3&4, the AUROC to differentiate DLB and PDD+ was 0.603 (0.451–0.754) by nested cross-validation, which indicated that gut microbiota could not efficiently differentiate DLB and PDD+. Taxonomic differences between DLB and PDD+ by ANCOM-BC and Wilcoxon rank sum test were indicated in Supplementary Table 7b. *Collinsella* was the only genera that was significantly increased in DLB compared to PDD+ by ANCOM-BC.

### Analysis of three genera in patients with or without cognitive decline

As shown above (Fig. 3b), three genera, *Ruminococcus torques*, *Bifidobacterium*, and *Collinsella,* were essential determinants to differentiate DLB and HY3&4. When relative abundances of the three genera were compared in controls (*n* = 147), PDD− at HY3&4 (*n* = 71), PDD+ at HY3&4 (*n* = 20), and DLB (*n* = 28), (i) *Ruminococcus torques* was increased in DLB compared to controls, (ii) *Bifidobacterium* was decreased in DLB compared to PDD−, and (iii) *Collinsella* was increased in DLB compared to controls (Fig. 3d–f). Thus, increased *Ruminococcus torques*, decreased *Bifidobacterium*, and increased *Collinsella* were unique to DLB.

### Correlation between five clinical features and bacterial abundances in DLB

We calculated Spearman’s rank correlation coefficients between five clinical features [age, disease duration, MMSE, total Movement Disorder Society’s (MDS) version of the Unified Parkinson’s Disease Rating Scale (UPDRS), MDS-UPDRS III] and the abundances of ten genera that were significantly changed in DLB compared to controls (Supplementary Table 8). *Ruminococcus torques* was negatively correlated with MMSE. *Eggerthella* and *Coprococcus* were positively and negatively correlated with total MDS-UPDRS, respectively. Thus, *Ruminococcus torques* was likely to be increased in dementia, whereas *Eggerthella* was likely to be increased and *Coprococcus* was likely to be decreased with the progression of parkinsonism in DLB. In contrast to DLB, neither of the three genera was significantly changed in PD in our meta-analysis of five countries^20^.

### Quantification of fecal bile acids

Three genera (*Ruminococcus torques*, *Collinsella*, and *Ruminococcus gnavus*), which had relative abundances of more than 0.5% in our cohort, carry 7β-hydroxysteroid dehydrogenase (7BHD) [EC 1.1.1.201] to catalyze bidirectional reactions between 7-ketolithocholic acid (7K-LCA) and ursodeoxycholic acid (UDCA) according to KEGG and UniRef90. We showed above that both *Ruminococcus torques* and *Collinsella* were high in DLB (Fig. 3d, f). *Ruminococcus gnavus* tended to be high in DLB and PDD+ at HY3&4 (Fig. 3g), which was similar to *Ruminococcus torques*. We quantified fecal UDCA and 7K-LCA concentrations, and calculated the ratio of UDCA/7K-LCA to estimate the activity of 7BHD. The UDCA/7K-LCA ratio was significantly increased in DLB compared to controls (Fig. 3h). The median of the UDCA/7K-LCA ratios was high in PDD− and PDD+ at HY3&4 compared to controls, but *p*-values were both greater than 0.999 (Fig. 3h). Spearman’s rank correlation coefficients between the UDCA/7K-LCA ratios and *Ruminococcus torques*, *Collinsella*, and *Ruminococcus gnavus* were −0.009 (*p* = 0.922), −0.189 (*p* = 0.036), and 0.396 (*p* < 0.0001), respectively.

### Comparison of four genera (*Ruminococcus torques, Bifidobacterium, Collinsella, and Ruminococcus gnavus*) between controls, iRBD, PD, and DLB

We additionally plotted the four genera (*Ruminococcus torques*, *Bifidobacterium*, *Collinsella*, and *Ruminococcus gnavus*) indicated in Fig. 3 in controls, iRBD, PD, and DLB (Supplementary Fig. 3). As we observed in the comparisons between DLB and HY3&4 (PDD− and PDD+) (Fig. 3d–g), *Ruminococcus torques*, *Collinsella*, and *Ruminococcus gnavus* were increased in DLB, and *Bifidobacterium* was increased in PD, although statistical significance was not always observed. In addition, the abundances of the four genera in iRBD were similar to those in controls.

## Discussion

We analyzed gut microbiota in DLB to examine whether any intestinal bacteria are unique to DLB, as well as to both DLB and PD with cognitive decline (PDD+). Analysis of each taxon between DLB and controls revealed that seven genera (*Agathobacter*, *Lachnospiraceae ND3007 group*, *Butyricicoccus*, *Coprococcus*, *Faecalibacterium*, *Fusicatenibacter*, and *Haemophilus*) were significantly decreased, and three genera (*Collinsella*, *Eggerthella*, and *Ruminococcus torques*) were significantly increased in DLB (Fig. 1 and Supplementary Table 2a). Correlation analysis of gut microbiota and clinical features in DLB revealed that decreased *Coprococcus* and increased *Eggerthella* were likely to be associated with the progression of parkinsonism, whereas increased *Ruminococcus torques* was likely to be associated with dementia (Supplementary Table 8). Six of the seven decreased genera excluding *Haemophilus* were SCFA-producing bacteria. Genera that were significantly decreased in DLB were similar to those in PD (Supplementary Tables 2a, 3a). Decreases of SCFA-producing bacteria have been repeatedly reported in PD^20,24,26^, Alzheimer’s disease^44–47^, and ALS^48,49^, and are likely to be a shared feature in neurodegenerative diseases. SCFA, especially butyrate, ameliorates mucosal inflammation and oxidative status, increases the intestinal mucin layer, and induces regulatory T cells by suppressing histone deacetylases^50–52^. Two of the three increased genera (*Collinsella* and *Ruminococcus torques*) were essential to differentiate DLB and HY3&4, and will be addressed later. To summarize, SCFA-producing genera were decreased in DLB, as has been observed in PD. In contrast, the three genera that were increased in DLB, were not changed in PD.

The overall composition of gut microbiota was significantly different in DLB compared to controls according to PERMANOVA (Table 2a). In addition, age, sex, and PPI, but not BMI, constipation, donepezil, or memantine, affected the overall composition of gut microbiota (Table 2b, c). The effects of age, sex, and PPI on gut microbiota have been previously reported: (i) aging decreases *Bifidobacterium*^53^, and increases *Bacteroides*, *Eubacterium*, and *Clostridiaceae*^54^; (ii) females have higher α-diversity of intestinal microbiota^55–57^; (iii) males show decreased *Bacteroides* and increased *Prevotella* in the Human Microbiome Project (HMP) Consortium^58^; and (iv) PPI increases *Streptococcus* and decreases *Faecalibacterium*^59^. Thus, the change in the overall composition of gut microbiota in DLB was also accounted for by the effects of age, sex, and PPI on specific bacteria.

As indicated in the introduction, α-synucleinopathies are comprised of iRBD, PD, and DLB, and more than 90% of iRBD patients later develop other forms of α-synucleinopathies^3^. DLB develops dementia first, whereas PDD+ develops dementia in the course of the progression of PD. PCoA showed that the centers of gravity were shifted with the progression of PD (Fig. 2a). PCoA is also consistent with the notion that iRBD is prodromal to PD and DLB. Clustering of the centers of gravity in DLB, HY3&4, and PDD+ prompted us to compare DLB vs HY3&4, as well as DLB vs PDD+ . Although random forest modeling failed to differentiate DLB and PDD+ , genus *Collinsella* was significantly increased in DLB compared to PDD+ (*q*-value by ANCOM-BC = 0.044, Supplementary Table 7b). On the other hand, random forest modeling to differentiate DLB and HY3&4 showed that three genera (*Ruminococcus torques*, *Bifidobacterium*, and *Collinsella*) were essential determinants (Fig. 3b).

*Ruminococcus torques* and *Collinsella* were both increased in DLB (Fig. 3d, f). *Bifidobacterium* will be discussed later. *Ruminococcus torques* is also increased in ulcerative colitis and Crohn’s disease^60^. *Ruminococcus torques* is the most efficient bacterium that degrades mucin 2 (*MUC2*), which constitutes the cell surface mucin in the colon^60^. *Collinsella* is also increased in rheumatoid arthritis^61^. *Collinsella* enhances gut permeability by decreasing the tight junction protein ZO-1 in a mouse model of rheumatoid arthritis^61^. *Collinsella* also increases the production of the proinflammatory cytokine IL-17A in human intestinal epithelial cell lines^61^. Increased *Ruminococcus torques* and *Collinsella* in DLB are thus likely to increase gut permeability. Increased gut permeability may cause exposure of the intestinal neural plexus to pesticides/herbicides and lipopolysaccharide (LPS), both of which are likely to predispose the neural plexus to oxidative stress and inflammation. Increased risks of PD by pesticides/herbicides have been repeatedly reported^62^. Increased intestinal permeability in PD has been demonstrated by decreased serum lipopolysaccharide (LPS)-binding protein^19,63^, as well as increased intestinal staining for nitrotyrosine and *E. coli*^63^. Pesticides/herbicides and LPS may potentiate the formation of abnormal α-synuclein fibrils in PD, and similar mechanisms may be operational in DLB.

*Collinsella* is also increased in atherosclerosis^64^ and coronary artery disease^65^, but its relevance to DLB remains unknown. In contrast to high *Collinsella* in DLB, rheumatoid arthritis^61^, atherosclerosis^64^, and coronary artery disease^65^, low *Collinsella* was associated with high mortality rates of COVID-19 in 953 healthy subjects in ten countries^66^. Indeed, *Collinsella* was low in patients with patients with COVID-19 in three reports^67–69^, although this observation was not confirmed in another report^70^. The reason for the apparently discordant effects of *Collinsella* on different diseases remains elusive.

We observed a statistically significant increase of the fecal UDCA/7K-LCA ratio only in DLB (Fig. 3h). *Ruminococcus torques*, *Collinsella*, and *Ruminococcus gnavus* are major intestinal bacteria carrying 7BHD (EC 1.1.1.201) that catalyzes bidirectional reactions between 7K-LCA and UDCA^71^. Interestingly, *Ruminococcus torques*, *Collinsella*, and *Ruminococcus gnavus* were ranked first, third, and sixth in recursive feature elimination to differentiate DLB and HY3&4 in random forest modeling (Supplementary Table 6). UDCA is a major secondary bile acid in the enterohepatic circulation^72^. UDCA suppresses pro-inflammatory cytokines like TNF-α, IL-1β, IL-2, IL-4, and IL-6^73,74^, and have anti-oxidant and anti-apoptotic effects^75,76^. UDCA and its taurine conjugate, tauroursodeoxycholate, inhibit Aβ-induced apoptosis and have mitochondrial protective effects in mouse models of Alzheimer’s disease^77–79^ and in fibroblasts derived from patients with Alzheimer’s disease^80^. The effects of UDCA on PD have also been repeatedly reported^81–83^. The increase of UDCA may mitigate inflammation-mediated dopaminergic cell death at the substantia nigra. In the neocortex, however, neuroinflammation may not critically trigger neuronal cell death, and suppression of neuroinflammation by UDCA may fail to mitigate the development of DLB. Indeed, intraperitoneal injection of LPS causes P_2_Y_6_ receptor-mediated activation of microglia and inflammatory neuronal loss in the substantia nigra, but not in the cortex or hippocampus^84^. Delayed neuronal cell death in the substantia nigra due to suppressed neuroinflammation in DLB masks the ɑ-synuclein pathology in the substantia nigra, which also accounts for the delayed age of onset of DLB compared to that of PD^6^.

In addition to *Ruminococcus torques* and *Collinsella, Eggerthella* was also increased in DLB compared to controls (Fig. 1, Supplementary Table 2a). Although *Eggerthella* does not have 7BHD (EC 1.1.1.201), *Eggerthella* also catalyzes secondary bile acids^85,86^. As *Eggerthella* inhibits inflammation in the gut by producing bile acids^85^, *Eggerthella* may have a similar effect as *Ruminococcus torques* and *Collinsella*.

We previously reported that increased *Lactobacillus* in PD was accounted for not by PD but by COMT inhibitors, drugs for PD^20^. Similarly, we here showed that increased *Bifidobacterium* in PD was accounted for not by PD but by COMT inhibitors (Supplementary Fig. 4a). *Bifidobacterium* was previously reported to be increased in PD in three meta-analyses including ours^20,87,88^, but the increase of *Bifidobacterium* might be due to COMT inhibitors. We showed that *Bifidobacterium* tended to be lower in PDD− compared to PDD+ (Fig. 3e). As the ratios of COMT inhibitor intake were not different between PDD− and PDD+ (*p* = 0.53 by Fisher’s exact test), the presence of dementia might have lowered *Bifidobacterium* in PDD+ . Similarly, the median of *Bifidobacterium* in DLB was lower than that in controls (*p* = 0.194 by Wilcoxon rank sum test, which became *p* > 0.999 after correcting for multiple comparisons in Fig. 3e), while nobody in DLB or controls was taking COMT inhibitors. In addition, *Bifidobacterium* was positively correlated with MMSE in patients with PD and DLB, who were not taking COMT inhibitors (Supplementary Fig. 4b). Thus, *Bifidobacterium* was likely to be increased by COMT inhibitors and decreased by dementia. Decreased *Bifidobacterium* is observed in Alzheimer’s disease^89,90^ and is predictive of rapid progression of non-motor symptoms including cognitive decline in PD^91^. Frequent coexistence of tauopathy in Alzheimer’s disease and DLB^92,93^ is also in accordance with the notion that *Bifidobacterium* is decreased in dementia. Administration of *Bifidobacterium* ameliorates cognitive dysfunction in a mouse model of Alzheimer’s disease^94,95^, as well as in humans^96,97^. Oral administration of *Bifidobacterium* elevates brain-derived neurotrophic factor (BDNF), a member of the neurotrophin family, in the brain of rodents^98^. BDNF plays a significant role in neurogenesis^99^ and is decreased in the autopsied brain of Alzheimer’s disease^100^. Similarly, decreased serum BDNF is related to the development of Alzheimer’s disease^101,102^ and the dopaminergic cell death in PD^102,103^. Serum BDNF, however, is paradoxically increased in Alzheimer’s disease^104^ and PD^105,106^, which is likely to represent compensatory mechanisms^104–106^. Thus, decreased *Bifidobacterium* in DLB and PDD+ may be causally associated with cognitive decline via decreased BDNF.

Our study has two limitations. First, the number of fecal samples of DLB patients was limited to 28, which disabled subgroup analysis, although this is a first report of gut microbiota in DLB. Second, we could not show whether the change of gut microbiota in DLB was the cause or the consequence. In future studies, more fecal samples of DLB patients and longitudinal analysis will be required.

In conclusion, similarly to PD, SCFA-producing genera were decreased in DLB. Additionally, *Ruminococcus torques* and *Collinsella* were increased in DLB, which were not changed in PD. High *Ruminococcus torques* and high *Collinsella*, which were predicted to increase intestinal permeability and to increase secondary bile acids, as well as low *Bifidobacterium*, which were observed in Alzheimer’s disease, were predictive of DLB in random forest models. Indeed, the production of UDCA was high in DLB, and increased UDCA in DLB may mitigate neuroinflammation at the substantia nigra. Therapeutic intervention to increase *Bifidobacterium* potentially retards the development and progression of DLB.

## Methods

### Patients

All studies were approved by the Ethical Review Committees of the Nagoya University Graduate School of Medicine (approval #2016-0151), Iwate Medical University (approval #H28-123), Okayama Kyokuto Hospital (approval #kyoIR-2016002), and Fukuoka University School of Medicine (approval #2016M027). We got written informed consent from all recruited subjects.

We obtained fecal samples in 224 PD patients, 26 iRBD patients, 28 DLB patients, and 147 controls (November 2016 to May 2019). DLB patients were diagnosed according to the Dementia with Lewy Bodies Consortium^5^. We excluded DLB patients with other chronic diseases including diabetes mellitus, heart failure, liver cirrhosis, malignancy, hematological diseases, and autoimmune diseases. Similarly, we excluded DLB patients who claimed to have taken antibiotics in the past one month.

### DNA isolation and 16S rRNA V3-V4 gene amplicon sequencing

The samples were transported from the participant’s home to Nagoya University below 4˚C, freeze-dried^107^, and subjected to DNA isolation and sequencing of the 16S rRNA V3–V4 region using a pair of primers (341F, 5′-CCTACGGGNGGCWGCAG-3′ and 805R, 5′-GACTACHVGGGTATCTAATCC-3′).^20,21^ Paired-end sequencing of 300-nucleotide fragments was performed using the MiSeq reagent kit V3 on a MiSeq System (Illumina). The 16S rRNA gene amplicon sequencing data were analyzed by QIIME2^108^ with DADA2 using the SILVA taxonomy database release 138^109,110^.

### Possible confounding factors

We compared six demographic and clinical features [age, sex, body mass index (BMI), constipation, proton pump inhibitor intake (PPI), and H_2_ blocker intake] between (i) controls and DLB, (ii) controls and PD, and (iii) controls and iRBD. Subjects with the stool frequency twice a week or less were defined to be constipated^111^.

We further analyzed the effects of (i) DLB, (ii) DLB, age, sex, BMI, constipation, and PPI, and (iii) donepezil and memantine in DLB patients, on the overall composition of gut microbiota with PERMANOVA^112^. All genera were included in this analysis. The effect of each feature was evaluated by three distance metrics of Chao^113^, unweighted-UniFrac^114^, and weighted-UniFrac^114^. Chao and unweighted/weighted-UniFrac were calculated with the R package vegan and QIIME2, respectively.

### Analysis of each taxon between (i) controls and DLB, (ii) controls and PD, (iii) DLB and HY3&4 (including both PDD− and PDD+), and (iv) DLB and PDD+ at any HY stages

Taxa were filtered at the genus and family levels using the following two conditions. First, for each taxon, we counted the number of samples in which the relative abundance of the taxon of interest was greater than 1E-4. The number of such samples should constitute more than 20% of all samples. Second, we chose taxa with the average relative abundance of more than 0.001.

For each pair of (i) controls and DLB, (ii) controls and PD, (iii) DLB and HY3&4 (including both PDD− and PDD+), and (iv) DLB and PDD+ at any HY stages, we tested the difference of each taxon using Analysis of Compositions of Microbiomes with Bias Correction (ANCOM-BC)^41^ and the Wilcoxon rank sum test. Five confounding factors (age, sex, BMI, constipation, and PPI) were included in the analysis with ANCOM-BC on R version 4.2.1. The Wilcoxon rank sum test was calculated with the mannwhitneyu functionality of scipy.stat on Python 3.8.2. The false discovery rate (FDR) by the Benjamini-Hochberg method^115^ of both ANCOM-BC and Wilcoxon rank sum test less than 0.05 was considered to be significant. We made random forest models using the RandomForestRegressor functionality of sklearn.ensemble on Python 3.8.2 to identify essential bacteria to differentiate each pair of (i), (ii), (iii), and (iv) by leave-one-out cross-validation, and to calculate AUROC by nested cross validation^22^.

### Analysis of the overall gut microbiota in controls, DLB, iRBD, PD at any HY stages, and PDD+ at any HY stages

PD patients with MMSE lower than 26^116^ were arbitrarily defined as PDD+ , as there is no definite criteria for PDD^4,117^. PD patients with MMSE ≥ 26 are indicated by PDD−. Microbiota data in controls, PD, and iRBD in our previous report^20,21^ were included in the overall analysis. We performed Principal Coordinates Analysis (PCoA) using controls, iRBD, PD at any HY stages, PDD+ at any HY stages, and DLB. Next, we performed *tmap*^43^, an integrative map based on topological data analysis for interpreting microbiome data and metadata simultaneously. Although we used all genera in this analysis, we plotted the 20 most abundant genera, as well as genera that were significantly changed in DLB. By *tmap*, we plotted bacteria, clinical features, and disease states on an identical two-dimensional plane and examined which features were close to each other.

### Quantification of fecal bile acids

The quantitative determination using a liquid chromatography with tandem mass spectrometry (LC-MS/MS) was performed to determined fecal concentrations of 7K-LCA and UDCA in 52 controls, 44 patients with HY3&4 (PDD− and PDD+), and 28 patients with DLB. 7K-LCA (> 97% purity) and UDCA (> 96% purity) were obtained from Tokyo Chemical Industry Co., Ltd. (Tokyo, Japan) and FUJIFILM Wako Pure Chemical Co. (Osaka, Japan), respectively. UDCA-D4 was purchased from Sigma Aldrich (St. Louis, MO, USA) and used as an internal standard.

Briefly, 20 mg of freeze-dried fecal samples were mixed with 1 ml of 70% ethanol and internal standard solution. After vigorous shaking and centrifugation, the supernatants were transferred into a solid phase extraction column (strong anion exchange column). The bile acids were eluted by 1 ml of 2% formic acid in acetonitrile. The eluates were injected into LC − MS/MS, which was composed of Agilent 1200 Infinity LC coupled with an Agilent Ultivo Triple Quadrupole LC/MS System (Agilent Technologies). The operating conditions of LC were as follows: LC column, Cadenza CD-C18 (Imtakt, Kyoto, Japan), 150 × 2 mm i.d., 3 μm silica; mobile phase A, H_2_O containing 5 mmol/l of formic acid; mobile phase B, 100 % of acetonitrile; and injection volume, 10 μL. A freeze-dried quality control (QC) sample was quantified every 20 freeze-dried samples. The precision of the QC was less than 5.4% relative standard deviation (%RSD).

## Supplementary information

SUPPLEMENTAL MATERIAL

## Data Availability

FASTQ files of our dataset are available at the DNA Data Bank of Japan (DDBJ) under the accession numbers of “DRA009229” for PD and controls and “DRA012438” for PD and conrols, and “DRA009322” for iRBD and “DRA011417” for iRBD.
