# Supplementary material for: Gut microbiota in dementia with Lewy bodies
Source: NPJ Parkinsons Dis. 2022 Dec 9;8:169. doi: 10.1038/s41531-022-00428-2 (PMC9734655; doi:10.1038/s41531-022-00428-2)
Supplement: Supplementary file 1 — SUPPLEMENTAL MATERIAL [file 41531_2022_428_MOESM1_ESM.pdf]

Supplementary Table 1. The number of PD patients at each Hoehn & Yahr (HY) stage with (PDD+) or without (PDD-) cognitive decline defined by MMSE < 26

HY stage	Number of samples	PDD+	PDD-
1	31 (13.8%)	0 (0.0%)	31 (100.0%)
2	99 (44.2%)	9 (9.1%)	90 (90.9%)
3	73 (32.6%)	14 (19.2%)	59 (80.8%)
4	18 (8.0%)	6 (33.3%)	12 (66.7%)
5	3 (1.3%)	2 (66.7%)	1 (33.3%)
Total	224 (100.0%)	31 (13.8%)	193 (86.2%)

Supplementary Table 2a. Comparison of genera between controls and DLB

Phylum	Class	Order	Family	Genus	*P*-value by ANCOM-BC	*Q*-value by ANCOM-BC	*P*-value by Wilcoxon rank sum test	*Q*-value by Wilcoxon rank sum test	±[a]	Average relative abundance
Firmicutes	*Clostridia*	*Lachnospirales*	*Lachnospiraceae*	*Agathobacter*	5.2E-06	4.2E-04*	1.9E-05	5.3E-04*	-	0.0175
Firmicutes	*Clostridia*	*Lachnospirales*	*Lachnospiraceae*	*Lachnospiraceae_ND3007_group*	1.0E-05	4.2E-04*	3.2E-04	3.8E-03*	-	0.0053
Firmicutes	*Clostridia*	*Oscillospirales*	*Butyricicoccaceae*	*Butyricicoccus*	2.6E-05	7.2E-04*	1.4E-06	1.2E-04*	-	0.0041
Firmicutes	*Clostridia*	*Lachnospirales*	*Lachnospiraceae*	*Coprococcus*	3.7E-05	7.8E-04*	0.011	0.044*	-	0.0063
Actinobacteriota	*Coriobacteriia*	*Coriobacteriales*	*Coriobacteriaceae*	*Collinsella*	1.7E-04	2.8E-03*	6.5E-03	0.032*	+	0.0267
Firmicutes	*Clostridia*	*Oscillospirales*	*Ruminococcaceae*	*Faecalibacterium*	5.8E-04	8.0E-03*	2.9E-06	1.2E-04*	-	0.0544
Actinobacteriota	*Coriobacteriia*	*Coriobacteriales*	*Eggerthellaceae*	*Eggerthella*	8.5E-04	9.9E-03*	1.0E-03	7.6E-03*	+	0.0023
Firmicutes	*Clostridia*	*Lachnospirales*	*Lachnospiraceae*	*Fusicatenibacter*	9.5E-04	9.9E-03*	4.9E-05	8.3-E04*	-	0.0187
Proteobacteria	*Gammaproteobacteria*	*Pasteurellales*	*Pasteurellaceae*	*Haemophilus*	1.1E-03	0.011*	5.0E-05	8.3-E04*	-	0.0011
Firmicutes	*Clostridia*	*Lachnospirales*	*Lachnospiraceae*	*Anaerostipes*	3.9E-03	0.032*	0.021	0.067	-	0.0146
Firmicutes	*Clostridia*	*Lachnospirales*	*Lachnospiraceae*	*[Ruminococcus]_torques_group*	6.3E-03	0.047*	9.6E-04	7.6E-03*	+	0.0168
Firmicutes	*Clostridia*	*Lachnospirales*	*Lachnospiraceae*	*[Eubacterium]_ventriosum_group*	0.015	0.10	6.9E-03	0.032*	-	0.0030
Firmicutes	*Clostridia*	*Lachnospirales*	*Lachnospiraceae*	*Lachnospira*	0.020	0.13	5.2E-04	5.4E-03*	-	0.0084
Firmicutes	*Clostridia*	*Peptostreptococcales-Tissierellales*	*Anaerovoracaceae*	*Family_XIII_AD3011_group*	0.023	0.14	8.6E-04	7.6E-03*	+	0.0013
Verrucomicrobiota	*Verrucomicrobiae*	*Verrucomicrobiales*	*Akkermansiaceae*	*Akkermansia*	0.026	0.14	1.7E-03	0.011*	+	0.0080
Firmicutes	*Clostridia*	*Lachnospirales*	*Lachnospiraceae*	*[Eubacterium]_eligens_group*	0.029	0.15	0.94	0.96	-	0.0038

Bacteroidota	*Bacteroidia*	*Bacteroidales*	*Prevotellaceae*	*Prevotella*	0.062	0.24	0.30	0.43	-	0.0291
Firmicutes	*Bacilli*	*Erysipelotrichales*	*Erysipelatoclostridiaceae*	*Erysipelatoclostridium*	0.060	0.24	0.031	0.093	+	0.0072
Firmicutes	*Bacilli*	*Erysipelotrichales*	*Erysipelotrichaceae*	*[Clostridium]_innocuum_group*	0.052	0.24	1.6E-03	0.011*	+	0.0018
Firmicutes	*Negativicutes*	*Acidaminococcales*	*Acidaminococcaceae*	*Phascolarctobacterium*	0.063	0.24	0.29	0.43	-	0.0087
Proteobacteria	*Gammaproteobacteria*	*Burkholderiales*	*Sutterellaceae*	*Sutterella*	0.054	0.24	0.13	0.25	-	0.0043
Proteobacteria	*Gammaproteobacteria*	*Enterobacterales*	*Enterobacteriaceae*	*Klebsiella*	0.058	0.24	0.019	0.067	+	0.0086
Firmicutes	*Clostridia*	*Lachnospirales*	*Lachnospiraceae*	*Lachnospiraceae_NK4A136_group*	0.069	0.25	0.021	0.067	-	0.0039
Firmicutes	*Clostridia*	*Christensenellales*	*Christensenellaceae*	*Christensenellaceae_R-7_group*	0.082	0.25	3.6E-03	0.020*	+	0.0058
Firmicutes	*Clostridia*	*Lachnospirales*	*Lachnospiraceae*	*Tyzzerella*	0.079	0.25	0.090	0.21	+	0.0056
Firmicutes	*Clostridia*	*Oscillospirales*	*Oscillospiraceae*	*UCG-005*	0.078	0.25	0.013	0.051	+	0.0035
Firmicutes	*Clostridia*	*Oscillospirales*	*Ruminococcaceae*	*uncultured*	0.080	0.25	3.2E-04	3.8E-03*	+	0.0054
Firmicutes	*Clostridia*	*Lachnospirales*	*Lachnospiraceae*	*[Ruminococcus]_gnavus_group*	0.097	0.28	0.50	0.61	+	0.0103
Firmicutes	*Clostridia*	*Lachnospirales*	*Lachnospiraceae*	*Lachnoclostridium*	0.095	0.28	0.13	0.25	+	0.0098
Firmicutes	*Bacilli*	*Lactobacillales*	*Lactobacillaceae*	*Lactobacillus*	0.14	0.38	4.0E-03	0.021*	+	0.0094
Bacteroidota	*Bacteroidia*	*Bacteroidales*	*Bacteroidaceae*	*Bacteroides*	0.15	0.38	0.020	0.067	-	0.2069
Firmicutes	*Bacilli*	*Erysipelotrichales*	*Erysipelatoclostridiaceae*	*Erysipelotrichaceae_UCG-003*	0.15	0.38	0.078	0.20	-	0.0083
Firmicutes	*Clostridia*	*Lachnospirales*	*Lachnospiraceae*	*Sellimonas*	0.15	0.38	0.12	0.24	+	0.0020
Firmicutes	*Clostridia*	*Oscillospirales*	*Oscillospiraceae*	*NK4A214_group*	0.18	0.43	0.049	0.13	+	0.0016
Firmicutes	*Clostridia*	*Peptostreptococcales-Tissierellales*	*Peptostreptococcaceae*	*Intestinibacter*	0.19	0.44	0.21	0.32	+	0.0055
Firmicutes	*Bacilli*	*Lactobacillales*	*Enterococcaceae*	*Enterococcus*	0.20	0.45	0.86	0.89	-	0.0024

Firmicutes	*Clostridia*	*Oscillospirales*	*Ruminococcaceae*	*CAG-352*	0.20	0.45	0.74	0.80	-	0.0169
Proteobacteria	*Gammaproteobacteria*	*Enterobacterales*	*Enterobacteriaceae*	*Enterobacter*	0.21	0.45	0.084	0.21	-	0.0023
Firmicutes	*Negativicutes*	*Veillonellales-Selenomonadales*	*Veillonellaceae*	*Dialister*	0.21	0.46	0.30	0.43	-	0.0034
Desulfobacterota	*Desulfovibrionia*	*Desulfovibrionales*	*Desulfovibrionaceae*	*Bilophila*	0.24	0.49	0.21	0.32	+	0.0011
Firmicutes	*Bacilli*	*Lactobacillales*	*Streptococcaceae*	*Streptococcus*	0.27	0.55	0.60	0.70	-	0.0378
Firmicutes	*Bacilli*	*Bacillales*	*Bacillaceae*	*Bacillus*	0.28	0.55	0.11	0.23	-	0.0014
Bacteroidota	*Bacteroidia*	*Bacteroidales*	*Marinifilaceae*	*Odoribacter*	0.30	0.56	0.21	0.32	-	0.0021
Firmicutes	*Clostridia*	*Clostridiales*	*Clostridiaceae*	*Clostridium_sensu_stricto_1*	0.30	0.56	0.12	0.24	+	0.0066
Firmicutes	*Clostridia*	*Oscillospirales*	*Ruminococcaceae*	*Subdoligranulum*	0.31	0.56	0.060	0.16	-	0.0243
Bacteroidota	*Bacteroidia*	*Bacteroidales*	*Barnesiellaceae*	*Coprobacter*	0.31	0.57	0.19	0.31	-	0.0012
Firmicutes	*Clostridia*	*Lachnospirales*	*Lachnospiraceae*		0.36	0.59	0.65	0.74	-	0.0113
Firmicutes	*Clostridia*	*Lachnospirales*	*Lachnospiraceae*	*Blautia*	0.36	0.59	0.29	0.43	-	0.0649
Firmicutes	*Clostridia*	*Lachnospirales*	*Lachnospiraceae*	*uncultured*	0.36	0.59	0.98	0.99	-	0.0113
Firmicutes	*Clostridia*	*Oscillospirales*	*Ruminococcaceae*		0.34	0.59	0.30	0.43	-	0.0054
Firmicutes	*Clostridia*	*Oscillospirales*	*Ruminococcaceae*	*[Eubacterium]_siraeum_group*	0.36	0.59	0.53	0.65	-	0.0011
Firmicutes	*Clostridia*	*Oscillospirales*	*Ruminococcaceae*	*Ruminococcus*	0.38	0.61	0.99	0.99	-	0.0119
Firmicutes	*Clostridia*	*Oscillospirales*	*Oscillospiraceae*	*Oscillibacter*	0.40	0.62	0.14	0.27	+	0.0017
Bacteroidota	*Bacteroidia*	*Bacteroidales*	*Tannerellaceae*	*Parabacteroides*	0.42	0.64	0.12	0.24	+	0.0191
Firmicutes	*Clostridia*	*Oscillospirales*	*Oscillospiraceae*	*uncultured*	0.42	0.64	2.7E-03	0.016*	+	0.0020
Firmicutes	*Bacilli*	*Erysipelotrichales*	*Erysipelotrichaceae*	*Turicibacter*	0.45	0.66	0.34	0.46	-	0.0022

Proteobacteria	*Gammaproteobacteria*	*Burkholderiales*	*Sutterellaceae*	*Parasutterella*	0.50	0.72	0.03	0.10	-	0.0019
Firmicutes	*Bacilli*	*Erysipelotrichales*	*Erysipelotrichaceae*	*Holdemanella*	0.52	0.74	0.59	0.70	+	0.0126
Bacteroidota	*Bacteroidia*	*Bacteroidales*	*Barnesiellaceae*	*Barnesiella*	0.53	0.74	0.73	0.80	+	0.0031
Firmicutes	*Clostridia*	*Clostridia UCG-014*	*Clostridia UCG-014*	*Clostridia UCG-014*	0.57	0.77	0.34	0.46	+	0.0031
Firmicutes	*Clostridia*	*Oscillospirales*	*Ruminococcaceae*	*Incertae_Sedis*	0.56	0.77	0.19	0.31	+	0.0045
Firmicutes	*Clostridia*	*Peptostreptococcales-Tissierellales*	*Peptostreptococcaceae*	*Romboutsia*	0.58	0.77	0.71	0.79	-	0.0091
Firmicutes	*Negativicutes*	*Veillonellales-Selenomonadales*	*Veillonellaceae*	*Megasphaera*	0.57	0.77	0.38	0.49	-	0.0041
Actinobacteriota	*Actinobacteria*	*Bifidobacteriales*	*Bifidobacteriaceae*	*Bifidobacterium*	0.67	0.83	0.19	0.31	+	0.0595
Firmicutes	*Clostridia*	*Lachnospirales*	*Lachnospiraceae*	*[Ruminococcus]_gauvreauii_group*	0.68	0.83	0.19	0.31	-	0.0041
Firmicutes	*Clostridia*	*Oscillospirales*	*Oscillospiraceae*	*Colidextribacter*	0.67	0.83	0.83	0.87	+	0.0012
Firmicutes	*Clostridia*	*Oscillospirales*	*Oscillospiraceae*	*Flavonifractor*	0.66	0.83	0.17	0.31	+	0.0014
Proteobacteria	*Gammaproteobacteria*	*Enterobacterales*	*Enterobacteriaceae*		0.65	0.83	0.58	0.70	+	0.0034
Firmicutes	*Clostridia*	*Peptostreptococcales-Tissierellales*	*Peptostreptococcaceae*	*Terrisporobacter*	0.71	0.84	0.45	0.58	+	0.0016
Fusobacteriota	*Fusobacteriia*	*Fusobacteriales*	*Fusobacteriaceae*	*Fusobacterium*	0.70	0.84	0.37	0.49	+	0.0056
Bacteroidota	*Bacteroidia*	*Bacteroidales*	*Prevotellaceae*	*Paraprevotella*	0.74	0.86	0.11	0.24	+	0.0026
Firmicutes	*Clostridia*	*Oscillospirales*	*[Eubacterium]_coprostanoligenes_group*	*[Eubacterium]_coprostanoligenes_group*	0.77	0.87	0.042	0.12	+	0.0109
Firmicutes	*Negativicutes*	*Veillonellales-Selenomonadales*	*Veillonellaceae*	*Veillonella*	0.77	0.87	0.015	0.058	-	0.0052
Firmicutes	*Clostridia*	*Lachnospirales*	*Lachnospiraceae*	*Roseburia*	0.79	0.87	0.024	0.074	+	0.0093

Firmicutes	*Clostridia*	*Oscillospirales*	*Oscillospiraceae*	*UCG-002*	0.78	0.87	0.77	0.83	+	0.0073
Proteobacteria	*Gammaproteobacteria*	*Enterobacterales*	*Enterobacteriaceae*	*Escherichia-Shigella*	0.82	0.89	0.10	0.22	+	0.0100
Bacteroidota	*Bacteroidia*	*Bacteroidales*	*Rikenellaceae*	*Alistipes*	0.85	0.91	0.16	0.30	+	0.0132
Firmicutes	*Clostridia*	*Monoglobales*	*Monoglobaceae*	*Monoglobus*	0.85	0.91	0.61	0.70	-	0.0041
Bacteroidota	*Bacteroidia*	*Bacteroidales*	*Muribaculaceae*	*Muribaculaceae*	0.89	0.93	0.83	0.87	+	0.0018
Firmicutes	*Negativicutes*	*Acidaminococcales*	*Acidaminococcaceae*	*Acidaminococcus*	0.90	0.94	0.38	0.49	-	0.0018
Bacteroidota	*Bacteroidia*	*Bacteroidales*	*Marinifilaceae*	*Butyricimonas*	0.94	0.95	0.46	0.58	-	0.0011
Firmicutes	*Clostridia*	*Lachnospirales*	*Lachnospiraceae*	*[Eubacterium]_hallii_group*	0.95	0.95	8.9E-03	0.039*	-	0.0116
Firmicutes	*Clostridia*	*Lachnospirales*	*Lachnospiraceae*	*Dorea*	0.94	0.95	0.10	0.22	-	0.0084

[a]Increased and decreased genera in DLB compared to controls are indicated by + and –, respectively.

Significantly different genera defined by *q*-values < 0.05 of both ANCOM-BC and Wilcoxon rank sum test are underlined.

**Q*-value less than 0.05

Supplementary Table 2b. Comparison of families between controls and DLB

Phylum	Class	Order	Family	*P*-value by ANCOM-BC	*Q*-value by ANCOM-BC	*P*-value by Wilcoxon rank sum test	*Q*-value by Wilcoxon rank sum test	±[a]	Average relative abundance
Actinobacteriota	*Coriobacteriia*	*Coriobacteriales*	*Eggerthellaceae*	2.0E-08	7.3E-07*	1.0E-07	3.6E-06*	+	0.0045
Desulfobacterota	*Desulfovibrionia*	*Desulfovibrionales*	*Desulfovibrionaceae*	2.7E-05	4.9E-04*	0.016	0.045*	+	0.0015
Actinobacteriota	*Coriobacteriia*	*Coriobacteriales*	*Coriobacteriaceae*	6.9E-05	6.2E-04*	5.2E-03	0.017*	+	0.0267
Firmicutes	*Clostridia*	*Peptostreptococcales-Tissierellales*	*Anaerovoracaceae*	5.5E-05	6.2E-04*	1.9E-05	2.3E-04*	+	0.0019
Firmicutes	*Clostridia*	*Oscillospirales*	*Ruminococcaceae*	1.9E-03	0.013*	2.1E-03	9.5E-03*	-	0.1232
Verrucomicrobiota	*Verrucomicrobiae*	*Verrucomicrobiales*	*Akkermansiaceae*	0.014	0.082	1.7E-03	8.8E-03*	+	0.0080
Firmicutes	*Clostridia*	*Christensenellales*	*Christensenellaceae*	0.037	0.19	1.6E-03	8.8E-03*	+	0.0060
Bacteroidota	*Bacteroidia*	*Bacteroidales*	*Prevotellaceae*	0.045	0.20	0.90	0.90	-	0.0378
Firmicutes	*Clostridia*	*Oscillospirales*	*Butyricicoccaceae*	0.051	0.20	2.3E-06	4.1E-05*	-	0.0043
Firmicutes	*Bacilli*	*Lactobacillales*	*Lactobacillaceae*	0.069	0.25	4.1E-03	0.015*	+	0.0096
Proteobacteria	*Gammaproteobacteria*	*Enterobacterales*	*Enterobacteriaceae*	0.083	0.27	1.2E-03	8.6E-03*	+	0.0256
Bacteroidota	*Bacteroidia*	*Bacteroidales*	*Bacteroidaceae*	0.17	0.36	2.0E-02	0.049*	-	0.2069
Bacteroidota	*Bacteroidia*	*Bacteroidales*	*Marinifilaceae*	0.19	0.36	0.84	0.88	+	0.0033
Bacteroidota	*Bacteroidia*	*Bacteroidales*	*Tannerellaceae*	0.19	0.36	0.078	0.15	+	0.0196
Firmicutes	*Bacilli*	*Erysipelotrichales*	*Erysipelotrichaceae*	0.16	0.36	0.059	0.12	+	0.0183
Firmicutes	*Clostridia*	*Clostridiales*	*Clostridiaceae*	0.13	0.36	0.12	0.20	+	0.0066
Firmicutes	*Clostridia*	*Lachnospirales*	*Lachnospiraceae*	0.17	0.36	0.048	0.10	-	0.2443
Firmicutes	*Clostridia*	*Monoglobales*	*Monoglobaceae*	0.17	0.36	0.61	0.70	+	0.0041

Firmicutes	*Clostridia*	*Oscillospirales*	*Oscillospiraceae*	0.15	0.36	0.017	0.045*	+	0.0200
Bacteroidota	*Bacteroidia*	*Bacteroidales*	*Muribaculaceae*	0.34	0.47	0.78	0.85	+	0.0018
Firmicutes	*Bacilli*	*Lactobacillales*	*Streptococcaceae*	0.29	0.47	0.67	0.75	-	0.0387
Firmicutes	*Clostridia*	*Clostridia UCG-014*	*Clostridia UCG-014*	0.29	0.47	0.34	0.45	+	0.0031
Firmicutes	*Clostridia*	*Oscillospirales*	*[Eubacterium]_coprostanoligenes_group*	0.34	0.47	0.042	0.095	+	0.0109
Firmicutes	*Clostridia*	*Peptostreptococcales-Tissierellales*	*Peptostreptococcaceae*	0.32	0.47	0.16	0.24	+	0.0163
Firmicutes	*Negativicutes*	*Acidaminococcales*	*Acidaminococcaceae*	0.32	0.47	0.098	0.18	-	0.0105
Firmicutes	*Negativicutes*	*Veillonellales-Selenomonadales*	*Selenomonadaceae*	0.31	0.47	0.57	0.68	+	0.0095
Bacteroidota	*Bacteroidia*	*Bacteroidales*	*Barnesiellaceae*	0.37	0.50	0.53	0.66	+	0.0045
Firmicutes	*Bacilli*	*Erysipelotrichales*	*Erysipelatoclostridiaceae*	0.40	0.52	0.13	0.20	+	0.0189
Fusobacteriota	*Fusobacteriia*	*Fusobacteriales*	*Fusobacteriaceae*	0.42	0.52	0.45	0.58	+	0.0058
Bacteroidota	*Bacteroidia*	*Bacteroidales*	*Rikenellaceae*	0.51	0.58	0.12	0.20	+	0.0138
Firmicutes	*Bacilli*	*Bacillales*	*Bacillaceae*	0.50	0.58	0.17	0.25	+	0.0014
Firmicutes	*Negativicutes*	*Veillonellales-Selenomonadales*	*Veillonellaceae*	0.49	0.58	9.8E-03	0.030*	-	0.0130
Actinobacteriota	*Actinobacteria*	*Bifidobacteriales*	*Bifidobacteriaceae*	0.58	0.64	0.20	0.28	+	0.0596
Proteobacteria	*Gammaproteobacteria*	*Burkholderiales*	*Sutterellaceae*	0.62	0.66	3.7E-03	0.015*	-	0.0061
Proteobacteria	*Gammaproteobacteria*	*Pasteurellales*	*Pasteurellaceae*	0.80	0.82	5.0E-05	4.5E-04*	-	0.0011
Firmicutes	*Bacilli*	*Lactobacillales*	*Enterococcaceae*	0.94	0.94	0.86	0.88	+	0.0024

[a]Increased and decreased families in DLB compared to controls are indicated by + and –, respectively.
Significantly different families defined by *q*-values < 0.05 of both ANCOM-BC and Wilcoxon rank sum test are underlined.
**Q*-value less than 0.05

Supplementary Table 3a. Comparison of genera between controls and PD

Phylum	Class	Order	Family	Genus	*P*-value by ANCOM-BC	*Q*-value by ANCOM-BC	*P*-value by Wilcoxon rank sum test	*Q*-value by Wilcoxon rank sum test	±[a]	Average relative abundance
Firmicutes	*Clostridia*	*Oscillospirales*	*Butyricicoccaceae*	*Butyricicoccus*	8.8E-08	7.4E-6[*]	2.9E-08	5.0E-07[*]	-	0.0033
Firmicutes	*Clostridia*	*Lachnospirales*	*Lachnospiraceae*	*Coprococcus*	1.3E-06	1.1E-04[*]	2.0E-04	9.1E-04[*]	-	0.0048
Firmicutes	*Clostridia*	*Lachnospirales*	*Lachnospiraceae*	*Blautia*	3.6E-06	3.0E-04[*]	5.0E-06	3.5E-05[*]	-	0.0550
Firmicutes	*Clostridia*	*Monoglobales*	*Monoglobaceae*	*Monoglobus*	1.2E-05	9.7E-04[*]	4.9E-04	1.8E-03[*]	-	0.0031
Firmicutes	*Clostridia*	*Lachnospirales*	*Lachnospiraceae*	*Fusicatenibacter*	2.5E-05	2.0E-03[*]	1.0E-05	6.2E-05[*]	-	0.0158
Firmicutes	*Clostridia*	*Lachnospirales*	*Lachnospiraceae*	*Lachnospiraceae_ND3007_group*	5.3E-05	4.2E-03[*]	6.7E-05	3.6E-04[*]	-	0.0044
Firmicutes	*Clostridia*	*Oscillospirales*	*Oscillospiraceae*	*Oscillibacter*	4.4E-04	0.032[*]	4.1E-08	5.9E-07[*]	+	0.0026
Firmicutes	*Clostridia*	*Lachnospirales*	*Lachnospiraceae*	*Agathobacter*	5.4E-04	0.038[*]	8.2E-06	5.4E-05[*]	-	0.0149
Verrucomicrobiota	*Verrucomicrobiae*	*Verrucomicrobiales*	*Akkermansiaceae*	*Akkermansia*	5.5E-04	0.039[*]	1.4E-04	6.4E-04[*]	+	0.0122
Firmicutes	*Clostridia*	*Christensenellales*	*Christensenellaceae*	*Christensenellaceae_R-7_group*	8.4E-04	0.058	4.0E-07	4.3E-06[*]	+	0.0082
Firmicutes	*Clostridia*	*Oscillospirales*	*Oscillospiraceae*	*uncultured*	9.5E-04	0.066	3.6E-09	7.7E-08[*]	+	0.0028
Bacteroidota	*Bacteroidia*	*Bacteroidales*	*Rikenellaceae*	*Alistipes*	1.5E-03	0.10	6.3E-07	5.9E-06[*]	+	0.0189
Firmicutes	*Clostridia*	*Lachnospirales*	*Lachnospiraceae*	*Lachnospira*	1.6E-03	0.11	6.1E-08	7.4E-07[*]	-	0.0065
Firmicutes	*Clostridia*	*Oscillospirales*	*Ruminococcaceae*	*uncultured*	1.7E-03	0.11	3.0E-10	8.5E-09[*]	+	0.0037
Bacteroidota	*Bacteroidia*	*Bacteroidales*	*Barnesiellaceae*	*Barnesiella*	1.8E-03	0.12	6.1E-03	0.016[*]	+	0.0047
Firmicutes	*Clostridia*	*Lachnospirales*	*Lachnospiraceae*	*Anaerostipes*	2.3E-03	0.15	6.3E-03	0.016[*]	-	0.0129
Firmicutes	*Clostridia*	*Peptostreptococcales-Tissierellales*	*Anaerovoracaceae*	*Family_XIII_AD3011_group*	2.3E-03	0.16	7.4E-11	3.1E-09[*]	+	0.0020

Firmicutes	*Clostridia*	*Oscillospirales*	*Oscillospiraceae*	*Colidextribacter*	3.1E-03	0.20	1.0E-04	5.0E-04*	+	0.0018
Firmicutes	*Clostridia*	*Oscillospirales*	*Ruminococcaceae*	*Faecalibacterium*	3.2E-03	0.21	4.8E-06	3.5-E05*	-	0.0487
Firmicutes	*Clostridia*	*Oscillospirales*	*Ruminococcaceae*	*Subdoligranulum*	4.0E-03	0.25	0.033	0.064	-	0.0229
Firmicutes	*Bacilli*	*Lactobacillales*	*Lactobacillaceae*	*Lactobacillus*	5.1E-03	0.32	3.0E-03	9.2E-03*	+	0.0175
Firmicutes	*Clostridia*	*Lachnospirales*	*Lachnospiraceae*	*[Ruminococcus]_gauvreauii_group*	9.7E-03	0.52	0.024	0.050*	-	0.0031
Firmicutes	*Clostridia*	*Peptostreptococcales-Tissierellales*	*Peptostreptococcaceae*	*Terrisporobacter*	0.011	0.56	0.45	0.59	-	0.0012
Firmicutes	*Clostridia*	*Lachnospirales*	*Lachnospiraceae*	*Roseburia*	0.016	0.72	0.018	0.040*	-	0.0075
Firmicutes	*Clostridia*	*Oscillospirales*	*Oscillospiraceae*	*UCG-005*	0.022	0.85	3.4E-04	1.3E-03*	+	0.0044
Actinobacteriota	*Actinobacteria*	*Bifidobacteriales*	*Bifidobacteriaceae*	*Bifidobacterium*	0.032	0.96	0.052	0.092	+	0.0697
Bacteroidota	*Bacteroidia*	*Bacteroidales*	*Tannerellaceae*	*Parabacteroides*	0.034	0.96	5.9E-03	0.016*	+	0.0199
Firmicutes	*Clostridia*	*Peptostreptococcales-Tissierellales*	*Peptostreptococcaceae*	*Intestinibacter*	0.043	0.96	0.12	0.19	-	0.0046
Firmicutes	*Bacilli*	*Erysipelotrichales*	*Erysipelatoclostridiaceae*	*Erysipelotrichaceae_UCG-003*	0.050	0.96	0.082	0.13	-	0.0077
Firmicutes	*Clostridia*	*Lachnospirales*	*Lachnospiraceae*	*[Eubacterium]_hallii_group*	0.061	0.96	0.20	0.29	-	0.0112
Firmicutes	*Clostridia*	*Oscillospirales*	*Ruminococcaceae*	*[Eubacterium]_siraeum_group*	0.062	0.96	0.51	0.62	-	0.0012
Firmicutes	*Clostridia*	*Oscillospirales*	*Oscillospiraceae*	*NK4A214_group*	0.066	0.96	9.5E-04	3.2E-03*	+	0.0024
Bacteroidota	*Bacteroidia*	*Bacteroidales*	*Bacteroidaceae*	*Bacteroides*	0.074	0.96	7.1E-03	0.017*	-	0.1976
Firmicutes	*Bacilli*	*Erysipelotrichales*	*Erysipelotrichaceae*	*Holdemanella*	0.074	0.96	0.023	0.048*	-	0.0098
Firmicutes	*Negativicutes*	*Veillonellales-Selenomonadales*	*Veillonellaceae*	*Dialister*	0.078	0.96	0.16	0.24	-	0.0028
Firmicutes	*Clostridia*	*Lachnospirales*	*Lachnospiraceae*	*Tyzzerella*	0.079	0.96	0.053	0.092	+	0.0057
Proteobacteria	*Gammaproteobacteria*	*Enterobacterales*	*Enterobacteriaceae*		0.079	0.96	0.80	0.86	-	0.0023

Proteobacteria	*Gammaproteobacteria*	*Enterobacterales*	*Enterobacteriaceae*	*Enterobacter*	0.082	0.96	0.65	0.76	-	0.0017
Firmicutes	*Bacilli*	*Erysipelotrichales*	*Erysipelotrichaceae*	*Faecalitalea*	0.083	0.96	5.5E-03	0.015*	+	0.0017
Firmicutes	*Clostridia*	*Clostridia vadinBB60 group*	*Clostridia vadinBB60 group*	*Clostridia_vadinBB60_group*	0.092	0.96	4.1E-05	2.3E-04*	+	0.0011
Firmicutes	*Clostridia*	*Oscillospirales*	*[Eubacterium]_coprostanoligenes_group*	*[Eubacterium]_coprostanoligenes_group*	0.11	0.96	3.1E-03	9.2E-03*	+	0.0130
Firmicutes	*Clostridia*	*Lachnospirales*	*Lachnospiraceae*		0.13	0.96	0.054	0.092	-	0.0100
Bacteroidota	*Bacteroidia*	*Bacteroidales*	*Barnesiellaceae*	*Coprobacter*	0.16	0.96	0.48	0.61	-	0.0014
Firmicutes	*Bacilli*	*Erysipelotrichales*	*Erysipelatoclostridiaceae*	*Erysipelatoclostridium*	0.16	0.96	7.2E-03	0.017*	+	0.0064
Firmicutes	*Clostridia*	*Lachnospirales*	*Lachnospiraceae*	*Dorea*	0.17	0.96	0.044	0.081	-	0.0080
Proteobacteria	*Gammaproteobacteria*	*Enterobacterales*	*Enterobacteriaceae*	*Klebsiella*	0.18	0.96	0.61	0.74	+	0.0043
Firmicutes	*Negativicutes*	*Veillonellales-Selenomonadales*	*Veillonellaceae*	*Veillonella*	0.18	0.96	0.011	0.026*	-	0.0043
Firmicutes	*Clostridia*	*Lachnospirales*	*Lachnospiraceae*	*Sellimonas*	0.19	0.96	2.9E-04	1.2E-03*	+	0.0026
Firmicutes	*Clostridia*	*Oscillospirales*	*Ruminococcaceae*		0.21	0.96	4.1E-06	3.5E-05*	+	0.0086
Firmicutes	*Clostridia*	*Lachnospirales*	*Lachnospiraceae*	*[Eubacterium]_eligens_group*	0.22	0.96	0.12	0.19	+	0.0049
Bacteroidota	*Bacteroidia*	*Bacteroidales*	*Muribaculaceae*	*Muribaculaceae*	0.23	0.96	0.44	0.59	-	0.0016
Firmicutes	*Clostridia*	*Lachnospirales*	*Lachnospiraceae*	*[Eubacterium]_ventriosum_group*	0.23	0.96	0.71	0.79	-	0.0031
Firmicutes	*Bacilli*	*Lactobacillales*	*Enterococcaceae*	*Enterococcus*	0.25	0.96	0.23	0.32	+	0.0042
Firmicutes	*Clostridia*	*Oscillospirales*	*Ruminococcaceae*	*Incertae_Sedis*	0.28	0.96	1.1E-03	3.5E-03*	+	0.0053
Firmicutes	*Clostridia*	*Clostridia UCG-014*	*Clostridia UCG-014*	*Clostridia_UCG-014*	0.30	0.96	0.019	0.041*	+	0.0048
Firmicutes	*Bacilli*	*Lactobacillales*	*Streptococcaceae*	*Streptococcus*	0.30	0.96	0.62	0.74	-	0.0365
Firmicutes	*Bacilli*	*Erysipelotrichales*	*Erysipelotrichaceae*	*Turicibacter*	0.30	0.96	0.073	0.12	+	0.0034

Firmicutes	*Bacilli*	*Bacillales*	*Bacillaceae*	*Bacillus*	0.32	0.96	0.51	0.62	-	0.0018
Fusobacteriota	*Fusobacteriia*	*Fusobacteriales*	*Fusobacteriaceae*	*Fusobacterium*	0.35	0.96	0.88	0.91	+	0.0052
Firmicutes	*Clostridia*	*Oscillospirales*	*Ruminococcaceae*	*UBA1819*	0.35	0.96	2.2E-11	1.9E-09*	+	0.0013
Proteobacteria	*Gammaproteobacteria*	*Burkholderiales*	*Sutterellaceae*	*Parasutterella*	0.35	0.96	0.91	0.93	-	0.0019
Bacteroidota	*Bacteroidia*	*Bacteroidales*	*Marinifilaceae*	*Odoribacter*	0.37	0.96	0.038	0.071	+	0.0031
Proteobacteria	*Gammaproteobacteria*	*Enterobacterales*	*Enterobacteriaceae*	*Escherichia-Shigella*	0.39	0.96	0.67	0.77	-	0.0077
Bacteroidota	*Bacteroidia*	*Bacteroidales*	*Prevotellaceae*	*Paraprevotella*	0.40	0.96	0.027	0.053	+	0.0029
Desulfobacterota	*Desulfovibrionia*	*Desulfovibrionales*	*Desulfovibrionaceae*	*Bilophila*	0.40	0.96	4.1E-03	0.012*	-	0.0012
Firmicutes	*Clostridia*	*Lachnospirales*	*Lachnospiraceae*	*Lachnoclostridium*	0.44	0.96	0.81	0.86	-	0.0090
Firmicutes	*Clostridia*	*Lachnospirales*	*Lachnospiraceae*	*[Ruminococcus]_gnavus_group*	0.53	0.96	0.92	0.93	+	0.0087
Firmicutes	*Clostridia*	*Lachnospirales*	*Lachnospiraceae*	*uncultured*	0.59	0.96	2.7E-04	1.2E-03*	+	0.0013
Firmicutes	*Bacilli*	*Erysipelotrichales*	*Erysipelotrichaceae*	*[Clostridium]_innocuum_group*	0.60	0.96	1.1E-03	3.5E-03*	-	0.0015
Bacteroidota	*Bacteroidia*	*Bacteroidales*	*Prevotellaceae*	*Prevotella*	0.61	0.96	0.19	0.28	-	0.0267
Firmicutes	*Clostridia*	*Peptostreptococcales-Tissierellales*	*Peptostreptococcaceae*	*Romboutsia*	0.63	0.96	0.85	0.90	+	0.0104
Firmicutes	*Clostridia*	*Lachnospirales*	*Lachnospiraceae*	*Lachnospiraceae_NK4A136_group*	0.66	0.96	0.73	0.81	-	0.0043
Firmicutes	*Clostridia*	*Oscillospirales*	*Oscillospiraceae*	*UCG-002*	0.71	0.96	0.090	0.14	+	0.0090
Actinobacteriota	*Coriobacteriia*	*Coriobacteriales*	*Eggerthellaceae*	*Eggerthella*	0.76	0.96	0.049	0.089	-	0.0022
Firmicutes	*Negativicutes*	*Acidaminococcales*	*Acidaminococcaceae*	*Acidaminococcus*	0.77	0.96	0.45	0.59	+	0.0020
Firmicutes	*Clostridia*	*Lachnospirales*	*Lachnospiraceae*	*[Ruminococcus]_torques_group*	0.80	0.96	0.44	0.59	+	0.0145

Firmicutes	*Clostridia*	*Oscillospirales*	*Oscillospiraceae*	*Flavonifractor*	0.80	0.96	0.014	0.032*	-	0.0016
Proteobacteria	*Gammaproteobacteria*	*Burkholderiales*	*Sutterellaceae*	*Sutterella*	0.82	0.96	0.47	0.61	+	0.0044
Bacteroidota	*Bacteroidia*	*Bacteroidales*	*Marinifilaceae*	*Butyricimonas*	0.83	0.96	7.8E-04	2.8E-03*	+	0.0015
Firmicutes	*Clostridia*	*Clostridiales*	*Clostridiaceae*	*Clostridium_sensu_stricto_1*	0.83	0.96	0.20	0.29	-	0.0069
Firmicutes	*Clostridia*	*Oscillospirales*	*Ruminococcaceae*	*Ruminococcus*	0.84	0.96	0.74	0.81	-	0.0123
Firmicutes	*Negativicutes*	*Acidaminococcales*	*Acidaminococcaceae*	*Phascolarctobacterium*	0.86	0.96	0.70	0.79	-	0.0088
Firmicutes	*Clostridia*	*Oscillospirales*	*Ruminococcaceae*	*CAG-352*	0.89	0.96	0.20	0.29	-	0.0192
Firmicutes	*Negativicutes*	*Veillonellales-Selenomonadales*	*Veillonellaceae*	*Megasphaera*	0.96	0.96	0.63	0.75	-	0.0043
Actinobacteriota	*Coriobacteriia*	*Coriobacteriales*	*Coriobacteriaceae*	*Collinsella*	0.96	0.96	0.94	0.94	-	0.0237

[a]Increased and decreased genera in PD compared to controls are indicated by + and –, respectively.
Significantly different genera defined by *q*-values < 0.05 of both ANCOM-BC and Wilcoxon rank sum test are underlined.
**Q*-value less than 0.05

Supplementary Table 3b. Comparison of families between controls and PD

Phylum	Class	Order	Family	*P*-value by ANCOM-BC	*Q*-value by ANCOM-BC	*P*-value by Wilcoxon rank sum test	*Q*-value by Wilcoxon rank sum test	± [a]	Average relative abundance
Firmicutes	*Clostridia*	*Lachnospirales*	*Lachnospiraceae*	4.9E-08	1.8E-06*	2.8E-09	3.4E-08*	-	0.216
Firmicutes	*Clostridia*	*Oscillospirales*	*Butyricicoccaceae*	1.2E-07	4.20E-06	9.1E-07	5.5E-06*	-	0.004
Firmicutes	*Clostridia*	*Monoglobales*	*Monoglobaceae*	2.6E-06	8.70E-05	4.9E-04	1.6E-03*	-	0.003
Firmicutes	*Clostridia*	*Oscillospirales*	*Oscillospiraceae*	1.2E-05	4.1E-04*	7.7E-10	1.4E-08*	+	0.026
Firmicutes	*Clostridia*	*Christensenellales*	*Christensenellaceae*	5.0E-04	0.016*	5.7E-08	5.2E-07*	+	0.009
Verrucomicrobiota	*Verrucomicrobiae*	*Verrucomicrobiales*	*Akkermansiaceae*	7.2E-04	0.022*	1.4E-04	4.9E-04*	+	0.012
Firmicutes	*Clostridia*	*Peptostreptococcales-Tissierellales*	*Anaerovoracaceae*	1.7E-03	0.049*	3.1E-11	1.1E-09*	+	0.003
Bacteroidota	*Bacteroidia*	*Bacteroidales*	*Rikenellaceae*	2.3E-03	0.068	4.8E-07	3.5E-06*	+	0.020
Firmicutes	*Bacilli*	*Lactobacillales*	*Lactobacillaceae*	6.7E-03	0.18	2.7E-03	7.1E-03*	+	0.018
Bacteroidota	*Bacteroidia*	*Bacteroidales*	*Barnesiellaceae*	0.011	0.28	4.6E-03	0.010*	+	0.007
Bacteroidota	*Bacteroidia*	*Bacteroidales*	*Tannerellaceae*	0.018	0.45	8.4E-04	2.3E-03*	+	0.021
Actinobacteriota	*Actinobacteria*	*Bifidobacteriales*	*Bifidobacteriaceae*	0.034	0.76	0.051	0.092	+	0.070
Bacteroidota	*Bacteroidia*	*Bacteroidales*	*Bacteroidaceae*	0.071	0.96	7.1E-03	0.015*	-	0.198
Bacteroidota	*Bacteroidia*	*Bacteroidales*	*Marinifilaceae*	0.12	0.96	6.3E-05	2.5E-04*	+	0.005
Firmicutes	*Clostridia*	*Oscillospirales*	*Ruminococcaceae*	0.13	0.96	0.24	0.33	-	0.126
Bacteroidota	*Bacteroidia*	*Bacteroidales*	*Muribaculaceae*	0.13	0.96	0.38	0.51	-	0.002

Firmicutes	*Clostridia*	*Oscillospirales*	*[Eubacterium]_coprostanoligenes_group*	0.14	0.96	3.1E-03	7.5E-03*	+	0.013
Firmicutes	*Bacilli*	*Bacillales*	*Bacillaceae*	0.16	0.96	0.40	0.51	-	0.002
Firmicutes	*Clostridia*	*Clostridia_vadinBB60_group*	*Clostridia_vadinBB60_group*	0.20	0.96	4.1E-05	1.9E-04*	+	0.001
Firmicutes	*Bacilli*	*Lactobacillales*	*Streptococcaceae*	0.22	0.96	0.69	0.75	-	0.037
Firmicutes	*Negativicutes*	*Veillonellales-Selenomonadales*	*Veillonellaceae*	0.25	0.96	0.010	0.021*	-	0.012
Firmicutes	*Bacilli*	*Erysipelotrichales*	*Erysipelatoclostridiaceae*	0.29	0.96	0.24	0.33	-	0.017
Firmicutes	*Bacilli*	*Lactobacillales*	*Enterococcaceae*	0.32	0.96	0.23	0.33	+	0.004
Firmicutes	*Clostridia*	*Clostridia_UCG-014*	*Clostridia_UCG-014*	0.39	0.96	0.019	0.037*	+	0.005
Fusobacteriota	*Fusobacteriia*	*Fusobacteriales*	*Fusobacteriaceae*	0.45	0.96	0.77	0.79	+	0.005
Proteobacteria	*Gammaproteobacteria*	*Enterobacterales*	*Enterobacteriaceae*	0.54	0.96	0.65	0.73	-	0.017
Firmicutes	*Clostridia*	*Peptostreptococcales-Tissierellales*	*Peptostreptococcaceae*	0.64	0.96	0.75	0.79	-	0.016
Firmicutes	*Clostridia*	*Clostridiales*	*Clostridiaceae*	0.73	0.96	0.21	0.33	-	0.007
Firmicutes	*Bacilli*	*Erysipelotrichales*	*Erysipelotrichaceae*	0.76	0.96	0.15	0.26	-	0.018
Bacteroidota	*Bacteroidia*	*Bacteroidales*	*Prevotellaceae*	0.78	0.96	0.23	0.33	-	0.036
Actinobacteriota	*Coriobacteriia*	*Coriobacteriales*	*Eggerthellaceae*	0.78	0.96	7.3E-04	2.2E-03*	-	0.004
Firmicutes	*Negativicutes*	*Acidaminococcales*	*Acidaminococcaceae*	0.81	0.96	0.65	0.73	+	0.011

Actinobacteriota	*Coriobacteriia*	*Coriobacteriales*	*Coriobacteriaceae*	0.91	0.96	1.00	1.00	-	0.024
Desulfobacterota	*Desulfovibrionia*	*Desulfovibrionales*	*Desulfovibrionaceae*	0.94	0.96	3.0E-05	1.6E-04*	+	0.002
Firmicutes	*Negativicutes*	*Veillonellales-Selenomonadales*	*Selenomonadaceae*	0.95	0.96	0.64	0.73	-	0.009
Proteobacteria	*Gammaproteobacteria*	*Burkholderiales*	*Sutterellaceae*	0.96	0.96	0.48	0.59	-	0.006

[a]Increased and decreased families in PD compared to controls are indicated by + and –, respectively.
Significantly different families defined by *q*-values < 0.05 of both ANCOM-BC and Wilcoxon rank sum test are underlined.
**Q*-value less than 0.05

Supplementary Table 4. Fifteen genera that most differentiated controls and DLB in random forest modeling in Supplementary Fig. 1

Phylum	Class	Order	Family	Genus	Feature importance
Firmicutes	*Clostridia*	*Peptostreptococcales-Tissierellales*	*Anaerovoracaceae*	*Family_XIII_AD3011_group*	0.220
Firmicutes	*Clostridia*	*Lachnospirales*	*Lachnospiraceae*	*Ruminococcus torques group*	0.088
Firmicutes	*Clostridia*	*Oscillospirales*	*Ruminococcaceae*	*uncultured*	0.079
Actinobacteriota	*Coriobacteriia*	*Coriobacteriales*	*Eggerthellaceae*	*Eggerthella*	0.077
Firmicutes	*Clostridia*	*Peptostreptococcales-Tissierellales*	*Peptostreptococcaceae*	*Intestinibacter*	0.067
Actinobacteriota	*Coriobacteriia*	*Coriobacteriales*	*Coriobacteriaceae*	*Collinsella*	0.063
Firmicutes	*Bacilli*	*Erysipelotrichales*	*Erysipelotrichaceae*	*[Clostridium]_innocuum_group*	0.054
Firmicutes	*Clostridia*	*Oscillospirales*	*Ruminococcaceae*	*Faecalibacterium*	0.052
Firmicutes	*Bacilli*	*Lactobacillales*	*Streptococcaceae*	*Streptococcus*	0.052
Firmicutes	*Clostridia*	*Lachnospirales*	*Lachnospiraceae*	*Coprococcus*	0.046
Proteobacteria	*Gammaproteobacteria*	*Enterobacterales*	*Enterobacteriaceae*	*Klebsiella*	0.046
Verrucomicrobiota	*Verrucomicrobiae*	*Verrucomicrobiales*	*Akkermansiaceae*	*Akkermansia*	0.046
Firmicutes	*Clostridia*	*Oscillospirales*	*Butyricicoccaceae*	*Butyricicoccus*	0.040
Bacteroidota	*Bacteroidia*	*Bacteroidales*	*Barnesiellaceae*	*Barnesiella*	0.036
Firmicutes	*Clostridia*	*Lachnospirales*	*Lachnospiraceae*	*Roseburia*	0.035

Genera that were significantly changed in DLB compared to controls are underlined (see Supplementary Table. 2a).

Supplementary Table 5. Twenty-five genera that differentiated controls and PD in random forest modeling in Supplementary Fig. 2

Phylum	Class	Order	Family	Genus	Feature importance
Firmicutes	*Clostridia*	*Oscillospirales*	*Ruminococcaceae*	*UBA1819*	0.102
Firmicutes	*Clostridia*	*Lachnospirales*	*Lachnospiraceae*	*Coprococcus*	0.069
Firmicutes	*Clostridia*	*Lachnospirales*	*Lachnospiraceae*	*Blautia*	0.060
Firmicutes	*Clostridia*	*Lachnospirales*	*Lachnospiraceae*	*Lachnospira*	0.053
Firmicutes	*Clostridia*	*Peptostreptococcales-Tissierellales*	*Anaerovoracaceae*	*Family_XIII_AD3011_group*	0.052
Firmicutes	*Bacilli*	*Lactobacillales*	*Lactobacillaceae*	*Lactobacillus*	0.043
Firmicutes	*Clostridia*	*Oscillospirales*	*Ruminococcaceae*	*uncultured*	0.039
Firmicutes	*Clostridia*	*Oscillospirales*	*Oscillospiraceae*	*uncultured*	0.038
Bacteroidota	*Bacteroidia*	*Bacteroidales*	*Bacteroidaceae*	*Bacteroides*	0.038
Firmicutes	*Clostridia*	*Lachnospirales*	*Lachnospiraceae*		0.037
Firmicutes	*Clostridia*	*Oscillospirales*	*Ruminococcaceae*	*Faecalibacterium*	0.037
Firmicutes	*Clostridia*	*Oscillospirales*	*Butyricicoccaceae*	*Butyricicoccus*	0.036
Firmicutes	*Clostridia*	*Lachnospirales*	*Lachnospiraceae*	*uncultured*	0.036
Bacteroidota	*Bacteroidia*	*Bacteroidales*	*Marinifilaceae*	*Odoribacter*	0.035
Firmicutes	*Clostridia*	*Lachnospirales*	*Lachnospiraceae*	*Agathobacter*	0.035
Actinobacteriota	*Actinobacteria*	*Bifidobacteriales*	*Bifidobacteriaceae*	*Bifidobacterium*	0.033
Firmicutes	*Clostridia*	*Oscillospirales*	*Oscillospiraceae*	*Oscillibacter*	0.031
Firmicutes	*Clostridia*	*Oscillospirales*	*Ruminococcaceae*	*Subdoligranulum*	0.030
Fusobacteriota	*Fusobacteriia*	*Fusobacteriales*	*Fusobacteriaceae*	*Fusobacterium*	0.029

Firmicutes	*Negativicutes*	*Acidaminococcales*	*Acidaminococcaceae*	*Phascolarctobacterium*	0.029
Firmicutes	*Clostridia*	*Oscillospirales*	*Oscillospiraceae*	*Flavonifractor*	0.029
Firmicutes	*Clostridia*	*Lachnospirales*	*Lachnospiraceae*	*[Eubacterium]_eligens_group*	0.027
Bacteroidota	*Bacteroidia*	*Bacteroidales*	*Rikenellaceae*	*Alistipes*	0.027
Firmicutes	*Clostridia*	*Oscillospirales*	*Ruminococcaceae*	*Incertae_sedis*	0.026
Firmicutes	*Clostridia*	*Peptostreptococcales-Tissierellales*	*Peptostreptococcaceae*	*Intestinibacter*	0.026

Genera that were significantly changed in PD compared to controls are underlined (see Supplementary Table 3a).

Supplementary Table 6. Top ten genera that differentiated DLB and HY3&4 (both PDD- and PDD+) in random forest modeling

Rank	Genus	AUROC
1	*Ruminococcus torques* *	0.738
2	*Bifidobacterium*	0.727
3	*Collinsella* *	0.825
4	*Blautia*	0.823
5	*Bacteroides*	0.807
6	*Ruminococcus gnavus* *	0.801
7	*Intestinibacter*	0.791
8	*Eubacterium hallii*	0.788
9	*Erysipelatoclostridium*	0.786
10	*Tyzzerella*	0.776

Area under the receiver operating characteristics curve (AUROC) by leave-one-out cross validation in recursive feature elimination is indicated. AUROC at the *N*th position was obtained when genera ranked 1st to *N*th were used for random forest modeling. For example, AUROC of 0.825 was obtained by *Ruminococcus torques*, *Bifidobacterium*, and *Collinsella*. AUROC values are plotted in Fig. 3b.

*Genera that carry 7-β-hydroxysteroid dehydrogenase (7BHD) to produce ursodeoxycholic acid.

Supplementary Table 7a. Comparison of genera between DLB and HY3&4 (both PDD- and PDD+)

Phylum	Class	Order	Family	Genus	*P*-value by ANCOM-BC	*Q*-value by ANCOM-BC	*P*-value by Wilcoxon rank sum test	*Q*-value by Wilcoxon rank sum test	± [a]
Firmicutes	*Bacilli*	*Erysipelotrichales*	*Erysipelotrichaceae*	*Turicibacter*	1.2E-03	0.059	0.19	0.54	-
Actinobacteriota	*Coriobacteriia*	*Coriobacteriales*	*Coriobacteriaceae*	*Collinsella*	1.8E-03	0.059	0.011	0.15	+
Firmicutes	*Clostridia*	*Lachnospirales*	*Lachnospiraceae*	*Blautia*	1.9E-03	0.059	3.2E-03	0.15	+
Firmicutes	*Clostridia*	*Lachnospirales*	*Lachnospiraceae*	*Ruminococcus torques*	3.2E-03	0.076	2.5E-03	0.15	+
Bacteroidota	*Bacteroidia*	*Bacteroidales*	*Marinifilaceae*	*Odoribacter*	5.3E-03	0.10	0.032	0.25	-
Firmicutes	*Clostridia*	*Oscillospirales*	*Ruminococcaceae*	*Fournierella*	8.4E-03	0.13	8.5E-03	0.15	-
Firmicutes	*Clostridia*	*Clostridia_vadinBB60_group*	*Clostridia_vadinBB60_group*	*Clostridia_vadinBB60_group*	0.011	0.15	0.067	0.32	-
Firmicutes	*Bacilli*	*Lactobacillales*	*Lactobacillaceae*	*Lactobacillus*	0.013	0.16	0.42	0.73	-
Firmicutes	*Clostridia*	*Oscillospirales*	*Ruminococcaceae*	*Negativibacillus*	0.016	0.16	0.045	0.28	-
Firmicutes	*Bacilli*	*Bacillales*	*Bacillaceae*	*Bacillus*	0.020	0.19	0.052	0.29	-
Firmicutes	*Clostridia*	*Lachnospirales*	*Lachnospiraceae*	*[Eubacterium]_eligens_group*	0.022	0.19	0.42	0.73	-
Firmicutes	*Bacilli*	*Erysipelotrichales*	*Erysipelotrichaceae*	*Faecalitalea*	0.034	0.26	7.1E-03	0.15	-
Actinobacteriota	*Coriobacteriia*	*Coriobacteriales*	*Eggerthellaceae*	*Eggerthella*	0.040	0.26	0.036	0.26	+
Firmicutes	*Bacilli*	*Erysipelotrichales*	*Erysipelotrichaceae*	*[Clostridium]_innocuum_group*	0.040	0.26	0.17	0.54	+
Firmicutes	*Clostridia*	*Oscillospirales*	*Oscillospiraceae*	*Colidextribacter*	0.042	0.26	0.039	0.26	-
Bacteroidota	*Bacteroidia*	*Bacteroidales*	*Barnesiellaceae*	*Coprobacter*	0.049	0.29	0.13	0.51	-
Firmicutes	*Clostridia*	*Clostridia_UCG-014*	*Clostridia_UCG-014*	*Clostridia_UCG-014*	0.053	0.29	0.29	0.61	-
Firmicutes	*Bacilli*	*Erysipelotrichales*	*Erysipelatoclostridiaceae*	*Erysipelatoclostridium*	0.060	0.30	0.28	0.60	+

Actinobacteriota	*Actinobacteria*	*Bifidobacteriales*	*Bifidobacteriaceae*	*Bifidobacterium*	0.066	0.30	7.0E-03	0.15	-
Firmicutes	*Clostridia*	*Oscillospirales*	*Ruminococcaceae*	*UBA1819*	0.066	0.30	0.14	0.51	+
Firmicutes	*Clostridia*	*Oscillospirales*	*Ruminococcaceae*	*Faecalibacterium*	0.067	0.30	0.031	0.25	-
Firmicutes	*Bacilli*	*Erysipelotrichales*	*Erysipelotrichaceae*	*Holdemanella*	0.073	0.31	0.019	0.19	+
Firmicutes	*Clostridia*	*Lachnospirales*	*Lachnospiraceae*	*Lachnoclostridium*	0.077	0.31	0.26	0.58	+
Firmicutes	*Clostridia*	*Oscillospirales*	*Ruminococcaceae*	*CAG-352*	0.083	0.32	0.56	0.79	-
Firmicutes	*Clostridia*	*Peptostreptococcales-Tissierellales*	*Peptostreptococcaceae*	*Romboutsia*	0.085	0.32	0.43	0.73	-
Firmicutes	*Clostridia*	*Oscillospirales*	*Oscillospiraceae*		0.090	0.32	0.10	0.45	-
Proteobacteria	*Gammaproteobacteria*	*Burkholderiales*	*Sutterellaceae*	*Parasutterella*	0.098	0.33	0.011	0.15	-
Firmicutes	*Clostridia*	*Lachnospirales*	*Lachnospiraceae*	*[Eubacterium]_ventriosum_group*	0.10	0.33	0.018	0.19	-
Firmicutes	*Clostridia*	*Clostridiales*	*Clostridiaceae*	*Clostridium_sensu_stricto_1*	0.11	0.36	0.83	0.89	-
Firmicutes	*Clostridia*	*Oscillospirales*	*UCG-010*	*UCG-010*	0.11	0.36	0.19	0.54	-
Firmicutes	*Bacilli*	*Lactobacillales*	*Enterococcaceae*	*Enterococcus*	0.12	0.36	0.13	0.51	-
Desulfobacterota	*Desulfovibrionia*	*Desulfovibrionales*	*Desulfovibrionaceae*	*Bilophila*	0.12	0.36	0.66	0.86	+
Bacteroidota	*Bacteroidia*	*Bacteroidales*	*Barnesiellaceae*	*Barnesiella*	0.14	0.39	0.18	0.54	-
Firmicutes	*Clostridia*	*Lachnospirales*	*Lachnospiraceae*	*Eisenbergiella*	0.15	0.39	0.065	0.32	+
Firmicutes	*Clostridia*	*Oscillospirales*	*Oscillospiraceae*	*uncultured*	0.15	0.39	0.80	0.88	-
Firmicutes	*Clostridia*	*Lachnospirales*	*Lachnospiraceae*	*[Ruminococcus]_gnavus_group*	0.15	0.39	0.20	0.54	+
Proteobacteria	*Gammaproteobacteria*	*Enterobacterales*	*Enterobacteriaceae*	*Klebsiella*	0.15	0.39	0.34	0.70	+
Firmicutes	*Clostridia*	*Lachnospirales*	*Lachnospiraceae*	*Agathobacter*	0.17	0.41	0.25	0.58	-
Bacteroidota	*Bacteroidia*	*Bacteroidales*	*Rikenellaceae*	*Alistipes*	0.17	0.41	0.21	0.55	-

Firmicutes	*Clostridia*	*Lachnospirales*	*Lachnospiraceae*	*Dorea*	0.18	0.41	0.57	0.79	+
Firmicutes	*Clostridia*	*Lachnospirales*	*Lachnospiraceae*	*Tyzzerella*	0.18	0.41	0.37	0.70	+
Firmicutes	*Clostridia*	*Oscillospirales*	*Ruminococcaceae*		0.19	0.42	0.061	0.32	-
Firmicutes	*Clostridia*	*Oscillospirales*	*[Eubacterium]_coprostanoligenes_group*	*[Eubacterium]_coprostanoligenes_group*	0.20	0.45	0.75	0.88	-
Firmicutes	*Clostridia*	*Oscillospirales*	*Oscillospiraceae*	*UCG-002*	0.25	0.51	0.23	0.56	-
Firmicutes	*Clostridia*	*Lachnospirales*	*Lachnospiraceae*	*Lachnospiraceae_ND3007_group*	0.25	0.51	0.79	0.88	-
Firmicutes	*Bacilli*	*Lactobacillales*	*Streptococcaceae*	*Lactococcus*	0.25	0.51	0.16	0.53	-
Firmicutes	*Clostridia*	*Oscillospirales*	*Ruminococcaceae*	*Ruminococcus*	0.26	0.51	0.68	0.87	-
Firmicutes	*Clostridia*	*Lachnospirales*	*Lachnospiraceae*	*Lachnospiraceae_NK4A136_group*	0.27	0.52	0.049	0.29	-
Firmicutes	*Negativicutes*	*Veillonellales-Selenomonadales*	*Veillonellaceae*	*Megasphaera*	0.28	0.52	0.73	0.88	+
Firmicutes	*Bacilli*	*Lactobacillales*	*Streptococcaceae*	*Streptococcus*	0.28	0.52	0.83	0.89	+
Firmicutes	*Clostridia*	*Monoglobales*	*Monoglobaceae*	*Monoglobus*	0.28	0.52	0.47	0.77	+
Firmicutes	*Clostridia*	*Lachnospirales*	*Lachnospiraceae*	*[Ruminococcus]_gauvreauii_group*	0.29	0.52	0.73	0.88	+
Proteobacteria	*Gammaproteobacteria*	*Enterobacterales*	*Enterobacteriaceae*		0.31	0.54	0.45	0.75	+
Firmicutes	*Negativicutes*	*Veillonellales-Selenomonadales*	*Veillonellaceae*	*Dialister*	0.31	0.54	0.89	0.92	+
Firmicutes	*Clostridia*	*Oscillospirales*	*Ruminococcaceae*	*Subdoligranulum*	0.35	0.59	0.49	0.77	+
Proteobacteria	*Gammaproteobacteria*	*Enterobacterales*	*Enterobacteriaceae*	*Escherichia-Shigella*	0.37	0.62	0.15	0.52	+
Verrucomicrobiota	*Verrucomicrobiae*	*Verrucomicrobiales*	*Akkermansiaceae*	*Akkermansia*	0.41	0.68	1.00	1.00	-
Proteobacteria	*Gammaproteobacteria*	*Burkholderiales*	*Sutterellaceae*	*Sutterella*	0.43	0.69	0.42	0.73	-
Firmicutes	*Clostridia*	*Lachnospirales*	*Lachnospiraceae*		0.44	0.70	0.40	0.73	-
Firmicutes	*Clostridia*	*Lachnospirales*	*Lachnospiraceae*	*Anaerostipes*	0.49	0.75	0.36	0.70	-

Firmicutes	*Clostridia*	*Peptostreptococcales-Tissierellales*	*Peptostreptococcaceae*	*Intestinibacter*	0.49	0.75	0.14	0.51	+
Firmicutes	*Negativicutes*	*Acidaminococcales*	*Acidaminococcaceae*	*Phascolarctobacterium*	0.49	0.75	0.77	0.88	-
Fusobacteriota	*Fusobacteriia*	*Fusobacteriales*	*Fusobacteriaceae*	*Fusobacterium*	0.51	0.77	0.022	0.21	+
Firmicutes	*Negativicutes*	*Acidaminococcales*	*Acidaminococcaceae*	*Acidaminococcus*	0.55	0.80	0.45	0.75	-
Firmicutes	*Clostridia*	*Lachnospirales*	*Lachnospiraceae*	*Sellimonas*	0.56	0.80	0.63	0.86	+
Bacteroidota	*Bacteroidia*	*Bacteroidales*	*Marinifilaceae*	*Butyricimonas*	0.58	0.80	0.20	0.54	-
Firmicutes	*Clostridia*	*Lachnospirales*	*Lachnospiraceae*	*Fusicatenibacter*	0.60	0.80	0.57	0.79	+
Firmicutes	*Clostridia*	*Christensenellales*	*Christensenellaceae*	*Christensenellaceae_R-7_group*	0.60	0.80	0.66	0.86	-
Firmicutes	*Clostridia*	*Eubacteriales*	*Eubacteriaceae*	*Eubacterium*	0.60	0.80	0.36	0.70	+
Proteobacteria	*Gammaproteobacteria*	*Enterobacterales*	*Enterobacteriaceae*	*Enterobacter*	0.60	0.80	0.19	0.54	-
Firmicutes	*Clostridia*	*Lachnospirales*	*Lachnospiraceae*	*Lachnospira*	0.62	0.80	0.86	0.91	+
Firmicutes	*Bacilli*	*Erysipelotrichales*	*Erysipelatoclostridiaceae*	*Erysipelotrichaceae_UCG-003*	0.62	0.80	0.55	0.79	+
Bacteroidota	*Bacteroidia*	*Bacteroidales*	*Muribaculaceae*	*Muribaculaceae*	0.62	0.80	0.36	0.70	+
Firmicutes	*Clostridia*	*Oscillospirales*	*Butyricicoccaceae*	*Butyricicoccus*	0.65	0.81	0.54	0.79	-
Firmicutes	*Clostridia*	*Lachnospirales*	*Lachnospiraceae*	*Coprococcus*	0.65	0.81	0.50	0.77	+
Firmicutes	*Clostridia*	*Lachnospirales*	*Lachnospiraceae*	*Roseburia*	0.67	0.83	0.49	0.77	+
Firmicutes	*Clostridia*	*Lachnospirales*	*Lachnospiraceae*	*uncultured*	0.68	0.83	0.20	0.54	-
Firmicutes	*Clostridia*	*Lachnospirales*	*Lachnospiraceae*	*Marvinbryantia*	0.69	0.83	0.56	0.79	+
Firmicutes	*Clostridia*	*Oscillospirales*	*Oscillospiraceae*	*UCG-005*	0.70	0.84	0.80	0.88	+
Firmicutes	*Clostridia*	*Oscillospirales*	*Oscillospiraceae*	*NK4A214_group*	0.72	0.85	0.69	0.87	-
Firmicutes	*Clostridia*	*Oscillospirales*	*Oscillospiraceae*	*Oscillibacter*	0.77	0.89	0.34	0.70	-

Firmicutes	*Clostridia*	*Oscillospirales*	*Ruminococcaceae*	*Incertae*	0.78	0.89	0.50	0.77	+
Bacteroidota	*Bacteroidia*	*Bacteroidales*	*Prevotellaceae*	*Prevotella*	0.78	0.89	0.66	0.86	-
Firmicutes	*Clostridia*	*Lachnospirales*	*Lachnospiraceae*	*[Eubacterium]_hallii_group*	0.82	0.91	0.092	0.41	+
Bacteroidota	*Bacteroidia*	*Bacteroidales*	*Bacteroidaceae*	*Bacteroides*	0.83	0.91	0.79	0.88	+
Firmicutes	*Clostridia*	*Oscillospirales*	*Ruminococcaceae*	*uncultured*	0.84	0.91	0.80	0.88	+
Bacteroidota	*Bacteroidia*	*Bacteroidales*	*Tannerellaceae*	*Parabacteroides*	0.85	0.92	0.69	0.87	+
Euryarchaeota	*Methanobacteria*	*Methanobacteriales*	*Methanobacteriaceae*	*Methanobrevibacter*	0.86	0.92	1.00	1.00	-
Firmicutes	*Clostridia*	*Oscillospirales*	*Oscillospiraceae*	*Flavonifractor*	0.87	0.92	0.94	0.96	+
Bacteroidota	*Bacteroidia*	*Bacteroidales*	*Tannerellaceae*		0.88	0.92	0.25	0.58	-
Bacteroidota	*Bacteroidia*	*Bacteroidales*	*Prevotellaceae*	*Paraprevotella*	0.90	0.92	0.76	0.88	+
Firmicutes	*Clostridia*	*Peptostreptococcales-Tissierellales*	*Anaerovoracaceae*	*Family_XIII_AD3011_group*	0.94	0.96	0.92	0.96	+
Firmicutes	*Negativicutes*	*Veillonellales-Selenomonadales*	*Veillonellaceae*	*Veillonella*	0.99	1.00	0.23	0.56	-
Firmicutes	*Clostridia*	*Peptostreptococcales-Tissierellales*	*Peptostreptococcaceae*	*Terrisporobacter*	1.00	1.00	0.79	0.88	+

[a]Increased and decreased genera in DLB compared to HY3&4 are indicated by + and –, respectively.
Three genera that made the maximum AUROC by leave-one-out cross validation in recursive feature elimination are underlined.

Supplementary Table 7b. Comparison of genera between DLB and PDD+ at any HY stages

Phylum	Class	Order	Family	Genus	*P*-value by ANCOM-BC	*Q*-value by ANCOM-BC	*P*-value by Wilcoxon rank sum test	*Q*-value by Wilcoxon rank sum test	± [a]
Actinobacteriota	*Coriobacteriia*	*Coriobacteriales*	*Coriobacteriaceae*	*Collinsella*	4.9E-04	0.044[*]	0.024	0.36	+
Firmicutes	*Clostridia*	*Lachnospirales*	*Lachnospiraceae*	*Blautia*	6.6E-03	0.24	0.088	0.61	+
Bacteroidota	*Bacteroidia*	*Bacteroidales*	*Marinifilaceae*	*Odoribacter*	0.013	0.24	7.1E-03	0.21	-
Firmicutes	*Bacilli*	*Erysipelotrichales*	*Erysipelotrichaceae*	*Turicibacter*	0.013	0.24	0.10	0.64	-
Firmicutes	*Clostridia*	*Oscillospirales*	*Oscillospiraceae*	*Colidextribacter*	0.013	0.24	6.9E-03	0.21	-
Firmicutes	*Clostridia*	*Lachnospirales*	*Lachnospiraceae*	*[Eubacterium]_eligens_group*	0.029	0.41	0.13	0.67	-
Proteobacteria	*Gammaproteobacteria*	*Enterobacterales*	*Enterobacteriaceae*	*Citrobacter*	0.036	0.41	1.4E-03	0.12	-
Firmicutes	*Clostridia*	*Clostridiales*	*Clostridiaceae*	*Clostridium_sensu_stricto_1*	0.037	0.41	0.29	0.78	-
Firmicutes	*Clostridia*	*Lachnospirales*	*Lachnospiraceae*	*Lachnoclostridium*	0.043	0.41	0.43	0.87	+
Firmicutes	*Bacilli*	*Erysipelotrichales*	*Erysipelotrichaceae*	*[Clostridium]_innocuum_group*	0.050	0.41	0.29	0.78	+
Firmicutes	*Clostridia*	*Clostridia_vadinBB60_group*	*Clostridia_vadinBB60_group*	*Clostridia_vadinBB60_group*	0.054	0.41	0.067	0.55	-
Bacteroidota	*Bacteroidia*	*Bacteroidales*	*Rikenellaceae*	*Alistipes*	0.057	0.41	0.033	0.42	-
Proteobacteria	*Gammaproteobacteria*	*Enterobacterales*	*Enterobacteriaceae*	*Klebsiella*	0.059	0.41	0.92	0.99	+
Firmicutes	*Clostridia*	*Lachnospirales*	*Lachnospiraceae*	*[Ruminococcus]_torques_group*	0.067	0.41	0.057	0.51	+
Firmicutes	*Clostridia*	*Peptostreptococcales-Tissierellales*	*Peptostreptococcaceae*	*Romboutsia*	0.070	0.41	0.48	0.87	-
Firmicutes	*Clostridia*	*Lachnospirales*	*Lachnospiraceae*	*Eisenbergiella*	0.072	0.41	0.078	0.59	+
Firmicutes	*Clostridia*	*Oscillospirales*	*[Eubacterium]_coprostanoligenes_group*	*[Eubacterium]_coprostanoligenes_group*	0.082	0.41	0.25	0.78	-

Firmicutes	*Bacilli*	*Erysipelotrichales*	*Erysipelotrichaceae*	*Faecalitalea*	0.082	0.41	0.021	0.36	-
Firmicutes	*Bacilli*	*Erysipelotrichales*	*Erysipelatoclostridiaceae*	*Erysipelatoclostridium*	0.091	0.41	0.92	0.99	+
Firmicutes	*Bacilli*	*Erysipelotrichales*	*Erysipelotrichaceae*	*Holdemanella*	0.092	0.41	0.11	0.64	+
Firmicutes	*Clostridia*	*Oscillospirales*	*Oscillospiraceae*		0.098	0.42	0.016	0.35	-
Firmicutes	*Clostridia*	*Lachnospirales*	*Lachnospiraceae*	*Dorea*	0.10	0.42	0.84	0.99	+
Firmicutes	*Clostridia*	*Christensenellales*	*Christensenellaceae*	*Christensenellaceae_R-7_group*	0.12	0.47	0.29	0.78	-
Firmicutes	*Clostridia*	*Oscillospirales*	*UCG-010*	*UCG-010*	0.13	0.48	0.17	0.67	-
Firmicutes	*Clostridia*	*Monoglobales*	*Monoglobaceae*	*Monoglobus*	0.14	0.50	0.87	0.99	+
Bacteroidota	*Bacteroidia*	*Bacteroidales*	*Barnesiellaceae*	*Coprobacter*	0.17	0.57	0.17	0.67	-
Firmicutes	*Clostridia*	*Lachnospirales*	*Lachnospiraceae*	*[Eubacterium]_ventriosum_group*	0.17	0.57	0.051	0.51	-
Firmicutes	*Bacilli*	*Lactobacillales*	*Lactobacillaceae*	*Lactobacillus*	0.18	0.57	0.56	0.87	-
Actinobacteriota	*Coriobacteriia*	*Coriobacteriales*	*Eggerthellaceae*	*Eggerthella*	0.18	0.57	0.12	0.67	+
Firmicutes	*Clostridia*	*Eubacteriales*	*Eubacteriaceae*	*Eubacterium*	0.20	0.60	0.82	0.99	+
Firmicutes	*Clostridia*	*Oscillospirales*	*Ruminococcaceae*	*UBA1819*	0.21	0.60	0.61	0.89	+
Firmicutes	*Clostridia*	*Lachnospirales*	*Lachnospiraceae*	*Anaerostipes*	0.21	0.60	0.34	0.79	+
Firmicutes	*Clostridia*	*Lachnospirales*	*Lachnospiraceae*	*Agathobacter*	0.22	0.60	0.56	0.87	-
Firmicutes	*Negativicutes*	*Veillonellales-Selenomonadales*	*Veillonellaceae*	*Veillonella*	0.24	0.60	0.90	0.99	+
Firmicutes	*Clostridia*	*Oscillospirales*	*Ruminococcaceae*	*Faecalibacterium*	0.24	0.60	0.14	0.67	-
Firmicutes	*Clostridia*	*Clostridia_UCG-014*	*Clostridia_UCG-014*	*Clostridia_UCG-014*	0.24	0.60	0.30	0.78	-
Firmicutes	*Negativicutes*	*Acidaminococcales*	*Acidaminococcaceae*	*Phascolarctobacterium*	0.25	0.60	0.28	0.78	-
Desulfobacterota	*Desulfovibrionia*	*Desulfovibrionales*	*Desulfovibrionaceae*	*Bilophila*	0.29	0.65	0.39	0.85	+

Firmicutes	*Clostridia*	*Lachnospirales*	*Lachnospiraceae*	*Lachnospiraceae_ND3007_group*	0.30	0.65	0.60	0.89	-
Proteobacteria	*Gammaproteobacteria*	*Enterobacterales*	*Enterobacteriaceae*	*Enterobacter*	0.31	0.65	0.85	0.99	-
Firmicutes	*Clostridia*	*Lachnospirales*	*Lachnospiraceae*	*NK4A136_group*	0.31	0.65	0.31	0.78	-
Verrucomicrobiota	*Verrucomicrobiae*	*Verrucomicrobiales*	*Akkermansiaceae*	*Akkermansia*	0.31	0.65	0.91	0.99	-
Firmicutes	*Clostridia*	*Oscillospirales*	*Oscillospiraceae*	*Lachnospiraceae_NK4A136_group*	0.31	0.65	0.17	0.67	-
Firmicutes	*Clostridia*	*Lachnospirales*	*Lachnospiraceae*	*Fusicatenibacter*	0.32	0.65	0.96	0.99	+
Bacteroidota	*Bacteroidia*	*Bacteroidales*	*Tannerellaceae*	*Parabacteroides*	0.32	0.65	0.52	0.87	+
Firmicutes	*Clostridia*	*Lachnospirales*	*Lachnospiraceae*		0.35	0.65	0.37	0.84	-
Actinobacteriota	*Actinobacteria*	*Actinomycetales*	*Actinomycetaceae*	*Actinomyces*	0.35	0.65	0.045	0.51	+
Bacteroidota	*Bacteroidia*	*Bacteroidales*	*Muribaculaceae*	*Muribaculaceae*	0.36	0.65	0.60	0.89	+
Firmicutes	*Clostridia*	*Oscillospirales*	*Ruminococcaceae*	*Ruminococcus*	0.36	0.65	0.54	0.87	-
Bacteroidota	*Bacteroidia*	*Bacteroidales*	*Tannerellaceae*		0.37	0.65	0.32	0.78	-
Bacteroidota	*Bacteroidia*	*Bacteroidales*	*Barnesiellaceae*	*Barnesiella*	0.37	0.65	0.32	0.78	-
Firmicutes	*Clostridia*	*Lachnospirales*	*Lachnospiraceae*	*[Ruminococcus]_gauvreauii_group*	0.38	0.66	0.56	0.87	+
Firmicutes	*Clostridia*	*Oscillospirales*	*Ruminococcaceae*	*uncultured*	0.40	0.67	0.54	0.87	+
Proteobacteria	*Gammaproteobacteria*	*Enterobacterales*	*Enterobacteriaceae*		0.43	0.71	0.25	0.78	+
Firmicutes	*Clostridia*	*Oscillospirales*	*Ruminococcaceae*	*CAG-352*	0.44	0.71	0.65	0.91	-
Firmicutes	*Clostridia*	*Lachnospirales*	*Lachnospiraceae*	*uncultured*	0.44	0.71	0.14	0.67	-
Firmicutes	*Clostridia*	*Oscillospirales*	*Oscillospiraceae*	*uncultured*	0.47	0.73	0.87	0.99	-
Euryarchaeota	*Methanobacteria*	*Methanobacteriales*	*Methanobacteriaceae*	*Methanobrevibacter*	0.47	0.73	0.53	0.87	-
Firmicutes	*Bacilli*	*Lactobacillales*	*Streptococcaceae*	*Streptococcus*	0.48	0.73	0.92	0.99	+

Firmicutes	*Bacilli*	*Lactobacillales*	*Enterococcaceae*	*Enterococcus*	0.50	0.74	0.53	0.87	-
Firmicutes	*Clostridia*	*Lachnospirales*	*Lachnospiraceae*	*Coprococcus*	0.51	0.76	0.84	0.99	+
Firmicutes	*Clostridia*	*Oscillospirales*	*Oscillospiraceae*	*Oscillibacter*	0.54	0.79	0.22	0.78	-
Firmicutes	*Clostridia*	*Lachnospirales*	*Lachnospiraceae*	*Tyzzerella*	0.55	0.79	0.75	0.94	+
Bacteroidota	*Bacteroidia*	*Bacteroidales*	*Prevotellaceae*	*Prevotella*	0.59	0.79	0.33	0.78	+
Firmicutes	*Clostridia*	*Oscillospirales*	*Oscillospiraceae*	*UCG-002*	0.60	0.79	0.65	0.91	-
Firmicutes	*Clostridia*	*Lachnospirales*	*Lachnospiraceae*	*[Eubacterium]_hallii_group*	0.60	0.79	0.26	0.78	+
Fusobacteriota	*Fusobacteriia*	*Fusobacteriales*	*Fusobacteriaceae*	*Fusobacterium*	0.60	0.79	0.29	0.78	-
Firmicutes	*Clostridia*	*Oscillospirales*	*Oscillospiraceae*	*UCG-005*	0.60	0.79	0.72	0.94	+
Firmicutes	*Bacilli*	*Erysipelotrichales*	*Erysipelatoclostridiaceae*	*Erysipelotrichaceae_UCG-003*	0.61	0.79	0.46	0.87	-
Firmicutes	*Clostridia*	*Peptostreptococcales-Tissierellales*	*Peptostreptococcaceae*	*Terrisporobacter*	0.62	0.80	0.98	0.99	+
Firmicutes	*Clostridia*	*Oscillospirales*	*Ruminococcaceae*	*[Eubacterium]_siraeum_group*	0.63	0.80	0.69	0.92	-
Firmicutes	*Clostridia*	*Oscillospirales*	*Oscillospiraceae*	*Flavonifractor*	0.68	0.84	0.66	0.91	-
Firmicutes	*Clostridia*	*Lachnospirales*	*Lachnospiraceae*	*Sellimonas*	0.68	0.84	0.68	0.91	+
Firmicutes	*Negativicutes*	*Veillonellales-Selenomonadales*	*Selenomonadaceae*	*Megamonas*	0.71	0.85	0.74	0.94	+
Proteobacteria	*Gammaproteobacteria*	*Burkholderiales*	*Sutterellaceae*	*Sutterella*	0.72	0.85	0.99	0.99	+
Firmicutes	*Clostridia*	*Lachnospirales*	*Lachnospiraceae*	*Roseburia*	0.72	0.85	0.46	0.87	+
Firmicutes	*Clostridia*	*Oscillospirales*	*Butyricicoccaceae*	*Butyricicoccus*	0.73	0.86	0.42	0.87	-
Firmicutes	*Clostridia*	*Peptostreptococcales-Tissierellales*	*Anaerovoracaceae*	*Family_XIII_AD3011_group*	0.76	0.87	0.64	0.91	+
Firmicutes	*Clostridia*	*Oscillospirales*	*Ruminococcaceae*	*Subdoligranulum*	0.77	0.87	0.68	0.91	+

Bacteroidota	*Bacteroidia*	*Bacteroidales*	*Marinifilaceae*	*Butyricimonas*	0.78	0.88	0.50	0.87	+
Firmicutes	*Clostridia*	*Oscillospirales*	*Ruminococcaceae*	*Incertae_Sedis*	0.80	0.88	0.41	0.87	+
Firmicutes	*Clostridia*	*Lachnospirales*	*Lachnospiraceae*	*Lachnospira*	0.80	0.88	0.49	0.87	-
Firmicutes	*Clostridia*	*Lachnospirales*	*Lachnospiraceae*	*[Ruminococcus]_gnavus_group*	0.83	0.90	0.99	0.99	+
Firmicutes	*Clostridia*	*Peptostreptococcales-Tissierellales*	*Peptostreptococcaceae*	*Intestinibacter*	0.87	0.92	0.74	0.94	-
Bacteroidota	*Bacteroidia*	*Bacteroidales*	*Prevotellaceae*	*Paraprevotella*	0.87	0.92	0.98	0.99	-
Actinobacteriota	*Actinobacteria*	*Bifidobacteriales*	*Bifidobacteriaceae*	*Bifidobacterium*	0.88	0.92	0.78	0.96	-
Proteobacteria	*Gammaproteobacteria*	*Enterobacterales*	*Enterobacteriaceae*	*Escherichia-Shigella*	0.89	0.92	0.28	0.78	+
Firmicutes	*Clostridia*	*Oscillospirales*	*Ruminococcaceae*		0.94	0.96	0.52	0.87	-
Firmicutes	*Clostridia*	*Christensenellales*	*Christensenellaceae*	*uncultured*	0.99	1.00	0.16	0.67	+
Bacteroidota	*Bacteroidia*	*Bacteroidales*	*Bacteroidaceae*	*Bacteroides*	1.00	1.00	0.96	0.99	+

[a]Increased and decreased genera in DLB compared to PDD+ are indicated by + and –, respectively.

Supplementary Table 8. Correlation analysis between five clinical features and ten genera that were significantly changed in DLB compared to controls.

Phylum	Class	Order	Family	Genus	Clinical feature	Spearman's correlation coefficient	*P*-value	*Q*-value
Firmicutes	*Clostridia*	*Oscillospirales*	*Butyricicoccaceae*	*Butyricicoccus*	Age	-0.32	0.10	0.74
Firmicutes	*Clostridia*	*Lachnospirales*	*Lachnospiraceae*	*Ruminococcus torques*	Age	-0.25	0.20	0.74
Actinobacteriota	*Coriobacteriia*	*Coriobacteriales*	*Eggerthellaceae*	*Eggerthella*	Age	0.18	0.36	0.74
Firmicutes	*Clostridia*	*Lachnospirales*	*Lachnospiraceae*	*Lachnospiraceae_ND3007_group*	Age	0.17	0.39	0.74
Actinobacteriota	*Coriobacteriia*	*Coriobacteriales*	*Coriobacteriaceae*	*Collinsella*	Age	-0.16	0.42	0.74
Proteobacteria	*Gammaproteobacteria*	*Pasteurellales*	*Pasteurellaceae*	*Haemophilus*	Age	0.14	0.48	0.74
Firmicutes	*Clostridia*	*Lachnospirales*	*Lachnospiraceae*	*Fusicatenibacter*	Age	-0.13	0.52	0.74
Firmicutes	*Clostridia*	*Lachnospirales*	*Lachnospiraceae*	*Agathobacter*	Age	0.050	0.80	0.89
Firmicutes	*Clostridia*	*Lachnospirales*	*Lachnospiraceae*	*Coprococcus*	Age	0.050	0.80	0.89
Firmicutes	*Clostridia*	*Oscillospirales*	*Ruminococcaceae*	*Faecalibacterium*	Age	0.014	0.94	0.94
Proteobacteria	*Gammaproteobacteria*	*Pasteurellales*	*Pasteurellaceae*	*Haemophilus*	Disease duration	0.40	0.037	0.37
Actinobacteriota	*Coriobacteriia*	*Coriobacteriales*	*Coriobacteriaceae*	*Collinsella*	Disease duration	-0.25	0.21	0.50
Firmicutes	*Clostridia*	*Oscillospirales*	*Butyricicoccaceae*	*Butyricicoccus*	Disease duration	0.24	0.22	0.50
Firmicutes	*Clostridia*	*Lachnospirales*	*Lachnospiraceae*	*Ruminococcu torques*	Disease duration	-0.23	0.24	0.50
Actinobacteriota	*Coriobacteriia*	*Coriobacteriales*	*Eggerthellaceae*	*Eggerthella*	Disease duration	-0.20	0.30	0.50
Firmicutes	*Clostridia*	*Oscillospirales*	*Ruminococcaceae*	*Faecalibacterium*	Disease duration	0.20	0.30	0.50
Firmicutes	*Clostridia*	*Lachnospirales*	*Lachnospiraceae*	*Fusicatenibacter*	Disease duration	0.17	0.38	0.54
Firmicutes	*Clostridia*	*Lachnospirales*	*Lachnospiraceae*	*Coprococcus*	Disease duration	-0.13	0.50	0.63

Firmicutes	*Clostridia*	*Lachnospirales*	*Lachnospiraceae*	*Agathobacter*	Disease duration	0.027	0.89	0.94
Firmicutes	*Clostridia*	*Lachnospirales*	*Lachnospiraceae*	*Lachnospiraceae_ND3007_group*	Disease duration	0.016	0.94	0.94
Firmicutes	*Clostridia*	*Lachnospirales*	*Lachnospiraceae*	*Ruminococcus torques*	MMSE	-0.62	7.0E-04	7.0E-03*
Firmicutes	*Clostridia*	*Oscillospirales*	*Ruminococcaceae*	*Faecalibacterium*	MMSE	0.34	0.085	0.43
Firmicutes	*Clostridia*	*Oscillospirales*	*Butyricicoccaceae*	*Butyricicoccus*	MMSE	0.30	0.14	0.47
Firmicutes	*Clostridia*	*Lachnospirales*	*Lachnospiraceae*	*Agathobacter*	MMSE	-0.22	0.27	0.50
Actinobacteriota	*Coriobacteriia*	*Coriobacteriales*	*Eggerthellaceae*	*Eggerthella*	MMSE	0.19	0.35	0.50
Firmicutes	*Clostridia*	*Lachnospirales*	*Lachnospiraceae*	*Coprococcus*	MMSE	-0.18	0.37	0.50
Actinobacteriota	*Coriobacteriia*	*Coriobacteriales*	*Coriobacteriaceae*	*Collinsella*	MMSE	-0.18	0.38	0.50
Proteobacteria	*Gammaproteobacteria*	*Pasteurellales*	*Pasteurellaceae*	*Haemophilus*	MMSE	-0.17	0.40	0.50
Firmicutes	*Clostridia*	*Lachnospirales*	*Lachnospiraceae*	*Fusicatenibacter*	MMSE	0.13	0.51	0.57
Firmicutes	*Clostridia*	*Lachnospirales*	*Lachnospiraceae*	*Lachnospiraceae_ND3007_group*	MMSE	0.081	0.69	0.69
Actinobacteriota	*Coriobacteriia*	*Coriobacteriales*	*Eggerthellaceae*	*Eggerthella*	Total MDS-UPDRS	0.525	4.1E-03	0.026*
Firmicutes	*Clostridia*	*Lachnospirales*	*Lachnospiraceae*	*Coprococcus*	Total MDS-UPDRS	-0.51	5.2E-03	0.026*
Firmicutes	*Clostridia*	*Oscillospirales*	*Ruminococcaceae*	*Faecalibacterium*	Total MDS-UPDRS	-0.34	0.081	0.21
Firmicutes	*Clostridia*	*Lachnospirales*	*Lachnospiraceae*	*Agathobacter*	Total MDS-UPDRS	-0.33	0.085	0.21
Firmicutes	*Clostridia*	*Lachnospirales*	*Lachnospiraceae*	*Fusicatenibacter*	Total MDS-UPDRS	-0.26	0.17	0.30
Firmicutes	*Clostridia*	*Oscillospirales*	*Butyricicoccaceae*	*Butyricicoccus*	Total MDS-UPDRS	-0.26	0.18	0.30
Proteobacteria	*Gammaproteobacteria*	*Pasteurellales*	*Pasteurellaceae*	*Haemophilus*	Total MDS-UPDRS	-0.22	0.27	0.39
Firmicutes	*Clostridia*	*Lachnospirales*	*Lachnospiraceae*	*Lachnospiraceae_ND3007_group*	Total MDS-UPDRS	-0.16	0.43	0.54
Firmicutes	*Clostridia*	*Lachnospirales*	*Lachnospiraceae*	*Ruminococcus torques*	Total MDS-UPDRS	-0.086	0.67	0.74

Actinobacteriota	*Coriobacteriia*	*Coriobacteriales*	*Coriobacteriaceae*	*Collinsella*	Total MDS-UPDRS	-0.041	0.83	0.83
Firmicutes	*Clostridia*	*Lachnospirales*	*Lachnospiraceae*	*Coprococcus*	MDS-UPDRS III	-0.51	6.1E-03	0.05
Actinobacteriota	*Coriobacteriia*	*Coriobacteriales*	*Eggerthellaceae*	*Eggerthella*	MDS-UPDRS III	0.48	0.010	0.05
Firmicutes	*Clostridia*	*Oscillospirales*	*Ruminococcaceae*	*Faecalibacterium*	MDS-UPDRS III	-0.36	0.062	0.21
Firmicutes	*Clostridia*	*Lachnospirales*	*Lachnospiraceae*	*Agathobacter*	MDS-UPDRS III	-0.30	0.11	0.25
Firmicutes	*Clostridia*	*Lachnospirales*	*Lachnospiraceae*	*Fusicatenibacter*	MDS-UPDRS III	-0.28	0.14	0.25
Firmicutes	*Clostridia*	*Oscillospirales*	*Butyricicoccaceae*	*Butyricicoccus*	MDS-UPDRS III	-0.28	0.15	0.25
Firmicutes	*Clostridia*	*Lachnospirales*	*Lachnospiraceae*	*Lachnospiraceae_ND3007_group*	MDS-UPDRS III	-0.25	0.20	0.29
Proteobacteria	*Gammaproteobacteria*	*Pasteurellales*	*Pasteurellaceae*	*Haemophilus*	MDS-UPDRS III	-0.17	0.38	0.48
Firmicutes	*Clostridia*	*Lachnospirales*	*Lachnospiraceae*	*Ruminococcus torques*	MDS-UPDRS III	-0.075	0.70	0.78
Actinobacteriota	*Coriobacteriia*	*Coriobacteriales*	*Coriobacteriaceae*	*Collinsella*	MDS-UPDRS III	0.018	0.93	0.93

Bacteria and clinical features with *q*-values less than 0.05 are underlined.

**Q*-value less than 0.05

Supplementary Figure 1. Random forest models and essential intestinal genera to differentiate controls and DLB

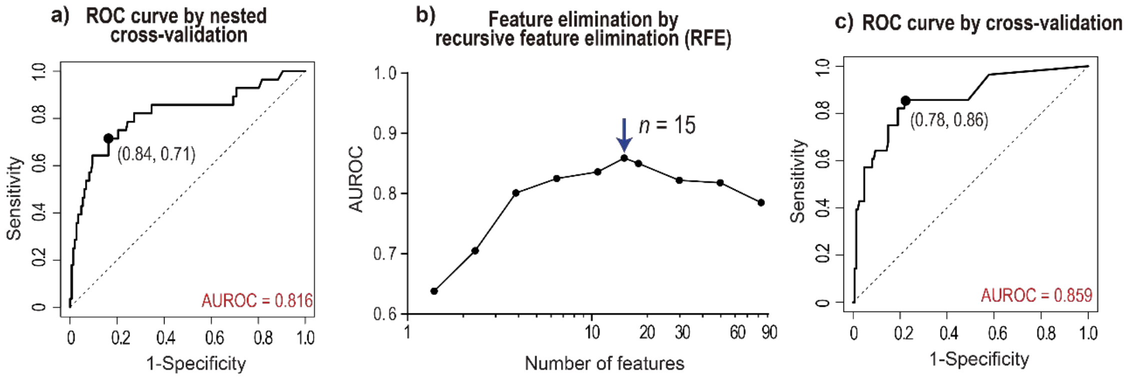

(a) ROC curve of nested cross-validation of random forest models to differentiate controls and DLB. The optimal point by Youden index is indicated by a dot with the specificity and sensitivity in parentheses. **(b)** A plot of the area under the receiver operating characteristics curve (AUROC) by leave-one-out cross-validation of random forest models, while genera (features) were recursively eliminated. An arrow points to the maximum AUROC with the number of genera. Fifteen genera that made the maximum AUROC are indicated in Supplementary Table 4. **(c)** ROC curve of leave-one-out cross-validation of random forest models generated with 15 genera indicated by an arrow in **(b)**. The optimal point by Youden index is indicated by a dot with the specificity and sensitivity in parentheses.

Supplementary Figure 2. Random forest models and essential intestinal genera to differentiate controls and PD

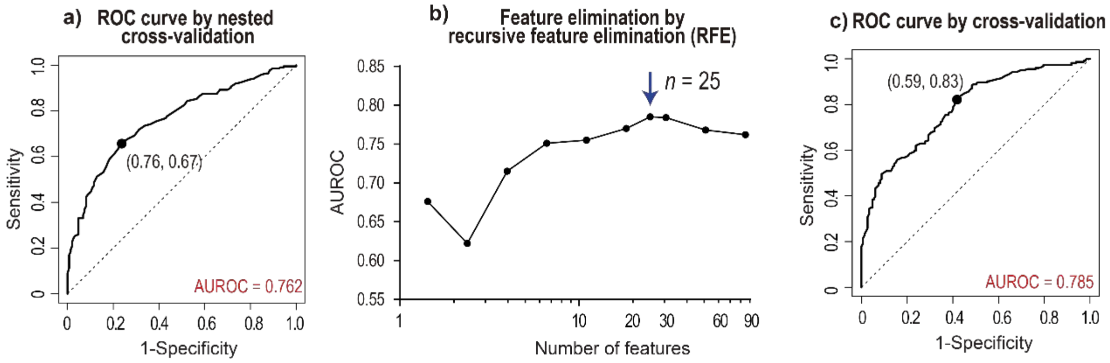

(a) ROC curves of nested cross-validation of random forest models to differentiate controls and PD. The optimal point by Youden index is indicated by a dot with the specificity and sensitivity in parentheses. **(b)** A plot of the area under the receiver operating characteristics curve (AUROC) by leave-one-out cross-validation of random forest models, while genera (features) were recursively eliminated. An arrow points to the maximum AUROC with the number of genera. Twenty-five genera that made the maximum AUROC are indicated in Supplementary Table 5. **(c)** ROC curves of leave-one-out cross-validation of random forest models generated with 25 genera indicated by an arrow in **(b)**. The optimal point by Youden index is indicated by a dot with the specificity and sensitivity in parentheses.

Supplementary Figure 3. Plots of relative abundances of four genera (*Ruminococcus torques*, *Bifidobacterium*, *Collinsella*, *and Ruminococcus gnavus*) in controls (*n* = 147), iRBD (*n* = 26), PD (*n* = 224), and DLB (*n* = 28)

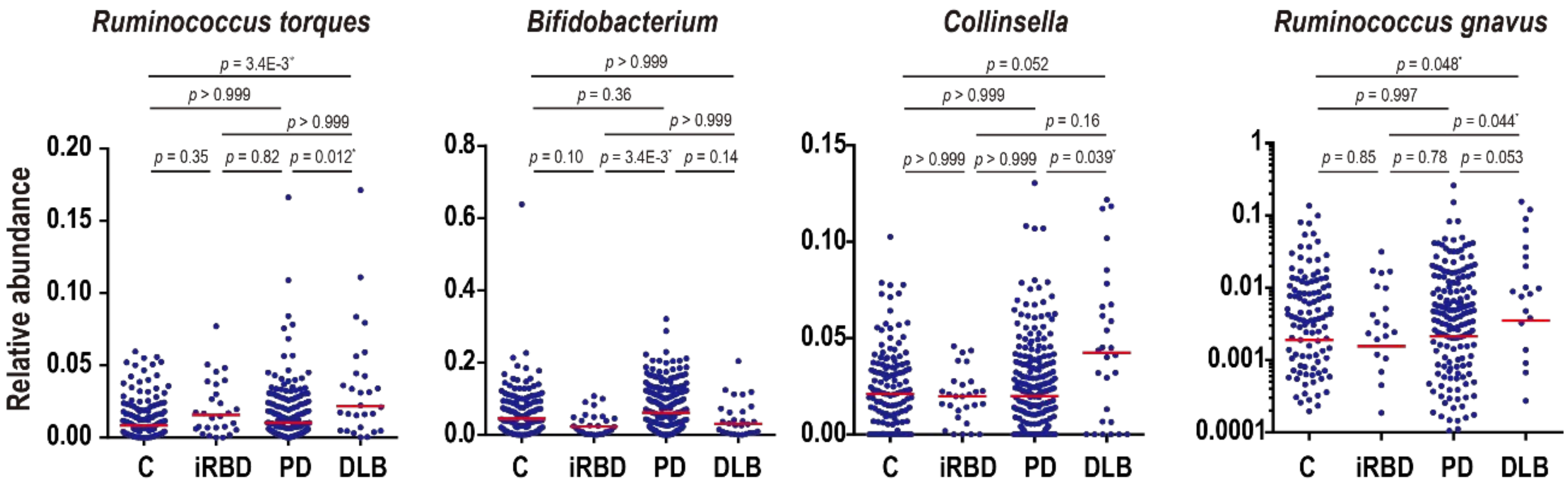

P-values by Kruskal-Wallis test were all less than 0.05. *P*-values by Dunn's post hoc test are indicated with an asterisk for $p < 0.05$.

Supplementary Figure 4. Relative abundances of *Bifidobacterium*

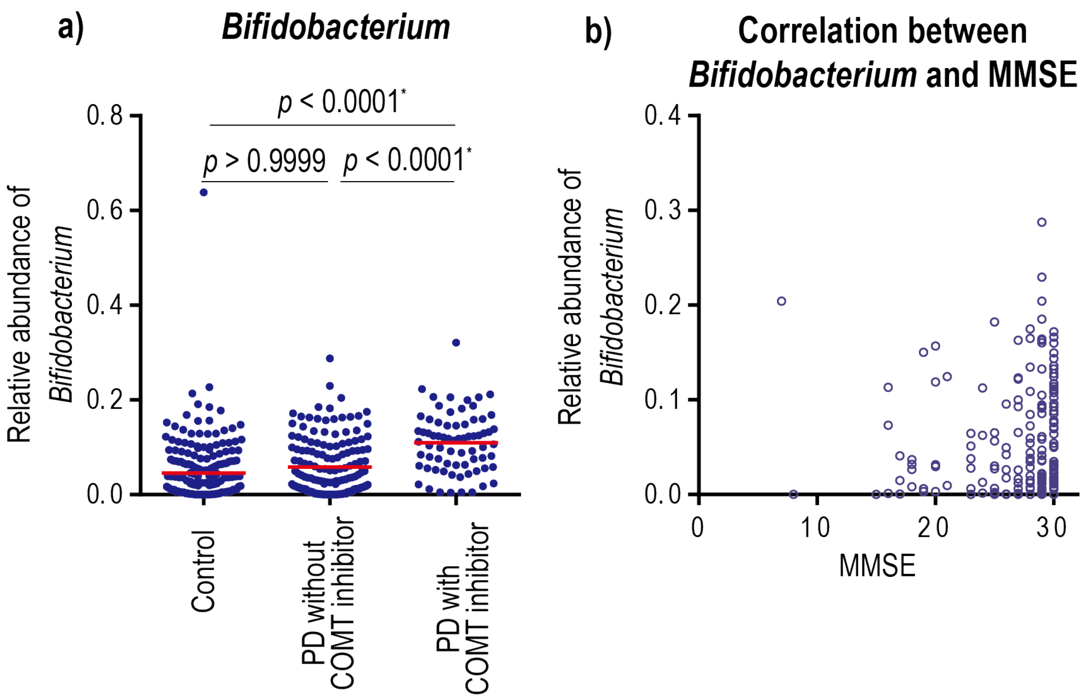

(a) Relative abundances of *Bifidobacterium* in controls (n = 147), PD without COMT inhibitor (n = 153), and PD with COMT inhibitor (n = 71). P-value by Kruskal-Wallis test was less than 0.05. P-values by Dunn's post hoc test are indicated with an asterisk for $p < 0.05$.

(b) Scatter plot of *Bifidobacterium* and Mini Mental State Examination (MMSE) in patients with PD and DLB, who were not taking COMT inhibitors. $\rho = 0.19$ and $p = 0.014$ by Spearman's rank correlation coefficient test.
